# Imaging therapeutic peptide transport across intestinal barriers

**DOI:** 10.1039/d1cb00024a

**Published:** 2021-06-15

**Authors:** Jannik Bruun Larsen, Nayere Taebnia, Alireza Dolatshahi-Pirouz, Anne Zebitz Eriksen, Claudia Hjørringgaard, Kasper Kristensen, Nanna Wichmann Larsen, Niels Bent Larsen, Rodolphe Marie, Ann-Kathrin Mündler, Ladan Parhamifar, Andrew James Urquhart, Arjen Weller, Kim I. Mortensen, Henrik Flyvbjerg, Thomas Lars Andresen

**Affiliations:** Center for Intestinal Absorption and Transport of Biopharmaceuticals, Department of Health Technology, Technical University of Denmark DK-2800, Kgs. Lyngby Denmark tlan@dtu.dk

## Abstract

Oral delivery is a highly preferred method for drug administration due to high patient compliance. However, oral administration is intrinsically challenging for pharmacologically interesting drug classes, in particular pharmaceutical peptides, due to the biological barriers associated with the gastrointestinal tract. In this review, we start by summarizing the pharmacological performance of several clinically relevant orally administrated therapeutic peptides, highlighting their low bioavailabilities. Thus, there is a strong need to increase the transport of peptide drugs across the intestinal barrier to realize future treatment needs and further development in the field. Currently, progress is hampered by a lack of understanding of transport mechanisms that govern intestinal absorption and transport of peptide drugs, including the effects of the permeability enhancers commonly used to mediate uptake. We describe how, for the past decades, mechanistic insights have predominantly been gained using functional assays with end-point read-out capabilities, which only allow indirect study of peptide transport mechanisms. We then focus on fluorescence imaging that, on the other hand, provides opportunities to directly visualize and thus follow peptide transport at high spatiotemporal resolution. Consequently, it may provide new and detailed mechanistic understanding of the interplay between the physicochemical properties of peptides and cellular processes; an interplay that determines the efficiency of transport. We review current methodology and state of the art in the field of fluorescence imaging to study intestinal barrier transport of peptides, and provide a comprehensive overview of the imaging-compatible *in vitro*, *ex vivo*, and *in vivo* platforms that currently are being developed to accelerate this emerging field of research.

## Abstract 2

Understanding how pharmaceutical peptides transport across the intestinal barrier could increase their bio-availability. To this end, fluorescence imaging offers a unique combination of spatiotemporal resolution and compatibility with living systems.

## Graphical abstract

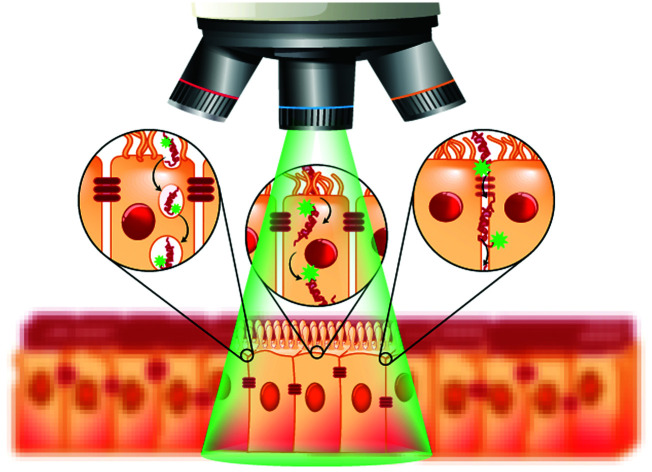

## 1. Introduction

Since the emergence of insulin therapy in the 1920s, peptides have been used extensively in medical practice.^1^ Peptides are ideal drug candidates, since they may disrupt protein–protein interaction efficiently and serve as ligands for cell-surface receptors.^1,2^ The worldwide market for peptide therapeutics has been estimated to more than double from 21.3 to 46.6 billion US$^1^ between 2015 and 2024. Typically, therapeutic peptides are administrated by injection,^3^ which limits the possibility for self-administration of the drug and lowers overall patient compliance.^4^ Therefore, alternative routes of delivery are areas of strong focus.^5^ Oral peptide delivery has received intense interest for decades.^6^ In this context, bio-availabilities of peptides above a few percent have proven extremely difficult to achieve, due to the biochemical and physical barriers presented by the gastric and the intestinal environment.^7^ The major obstacles include enzymatic peptide degradation and poor absorption through the epithelial cell layer.^8^ Consequently, considerable efforts have been devoted to the development of various delivery systems and permeation enhancers (PEs), such as fatty acids, surfactants, and bile salts.^5^ However, clinically approved delivery strategies for uptake *via* the gastro-intestinal tract remain scarce.^1,3,7^

**Fig. 1:**
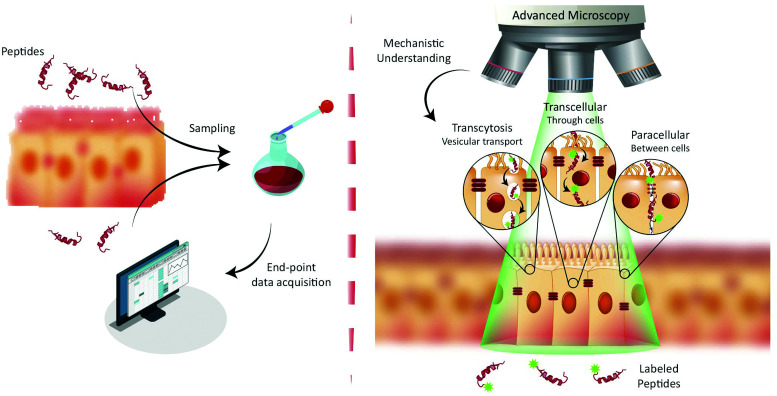
Paradigms for studying peptide transport across intestinal barriers. (left) Traditionally, peptide transport across intestinal barriers has been studied using barrier model systems employing end-point assays offering only indirect mechanistic insight on peptide transport mechanisms. (right) Fluorescence live-cell imaging offers the ability to directly visualize and track peptide transport across intestinal barrier models allowing for determination of the transport mechanisms (such as transcytosis, transcellular and/or paracellular) governing peptide transport.

In the quest for new peptide drugs and delivery systems, the quality of potential candidates is typically assessed using end-point bio-availability measurements (Fig. 1, left).^8^ Positive hits are identified by their increased transport across experimental models of the physiological and cellular barriers of the intestine.^9^ Such model systems span a vast range of technical and biological complexities; they range from simple artificial membranes to tissue samples.^8^ While the model systems do permit the quantification of pharmaceutical peptide transport across a biological barrier, they typically do not yield information about which cellular mechanisms that facilitated translocation. This approach was used, *e.g.*, in the development of the previously described array of PEs. Thus, many constructs and strategies have been tested, but their full biological mechanisms of action are complex and remain to be fully elucidated, which has led to concerns about the long-term use of PEs in chronic administration.^10,11^

The end-point screening methods applied for development of peptide drug candidates have recently been challenged by a community that has realized the importance of understanding the biological mechanisms governing drug delivery.^9,12^ For transport studies on peptide translocation across the intestinal barrier, seminal mechanistic efforts focused on using pharmacological agents to modify tight junction (TJ) integrity or selectively disrupt endocytosis pathways.^8,13^ Such studies have helped elucidate the major translocation pathways of the intestinal barrier employed by orally administrated small-molecule drugs and peptides.^1^ These pathways include (Fig. 1, right) active transcytosis mediated vesicular transport, passive transcellular transport through epithelial cells, and paracellular transport between epithelial cells through the TJs. Despite the widespread use of pharmacological pathway inhibitors, their specificity has been disputed, as they have been shown to affect multiple endocytosis pathways simultaneously.^14–16^ Therefore, more direct, rigorous mechanistic insight into peptide transport has been sought through other routes, most noticeably through the employment of fluorescence imaging.^13,17,18^ The great advantage of this approach is the ability to directly track peptide transport in live-cell setups involving only a minimal disturbance of the cells’ natural milieu (Fig. 1, right). Image-based transport studies should provide direct mechanistic information about the absorption and transport of peptides through and across the intestinal barriers. So far fluorescence-based methods have only routinely been used to study peptide transport in individual cells, as it is non-trivial to combine live-cell imaging with more realistic models of cellular barriers. However recent, significant strides in the field provide new and exciting opportunities for mechanistic studies of peptide transport across cellular barriers using more complex experimental setups and imaging modalities. The increase in mechanistic information should foster rationally design modifications to peptides and their delivery systems. Such guided modifications might optimize the bio-availability and end-point efficacy of orally delivered peptide pharmaceuticals dramatically.

Here, we briefly review what is known mechanistically about the modes of action of the clinically approved oral peptide and PE formulations as well as how conventional fluorescence imaging has aided to these ends. We then consider all steps involved in design and implementation of fluorescence image-based peptide-translocation studies: first, we discuss the choice of fluorescence imaging modality, the chemical considerations regarding choice of fluorescent probe and site of modification, and the biophysical characterization techniques used to monitor peptide stability and membrane interaction. Next we discuss the range of *in vitro* and *ex vivo* barrier models that are currently being developed to facilitate image-based studies and how *in vivo* imaging studies are emerging as an important method towards understanding peptide transport in the native environment. Finally, we briefly review the insights gained on how nanoparticle (NP) delivery systems made from peptides translocate across cellular barriers using imaging-based platforms.

## 2. Oral peptide drugs for systemic applications used in the clinic

Of all the classical non-invasive delivery routes, orally administrated pharmaceutical peptides had the largest share of clinical trials in 2019, emphasizing the strong effort in translating oral drugs to the clinic.^19^ Nevertheless, only four peptide treatments designed for transport across the intestinal barrier have yet been approved by the FDA.^20^ Despite earlier success with oral dosage forms of Cyclosporin A (CsA) and desmopressin in the 1980s, it has proven difficult to push other oral peptide drugs into the clinic. In fact, no other peptide drug for oral administration progressed beyond Phase II trials between 1987–2010.^21^ Only very recently, did the field experience a resurgence with the FDA approval of oral semaglutide in 2019 and octreotide in 2020. The historical development of oral peptide drugs and the current status of candidates in end-stage clinical trials have been reviewed extensively recently.^5,21,22^ Here we instead focus on the four peptide drugs currently approved for use in the clinic. We focus on the mechanisms involved in their successful transport across cell barriers and discuss instances where fluorescence imaging has helped elucidate these mechanisms.

### 2.1 Cyclosporin A

CsA is a cyclic undecapeptide used as an immunosuppressant to treat graft-versus-host disease in transplant patients. It was approved by the FDA for clinical use in 1983.^23^ The case of CsA is unique due to its relatively high bioavailability (BA) (above 30%), which has spurred in-depth mechanistic studies aimed at elucidating the key structural features promoting the passive diffusion of CsA across cellular barriers.^24^ Based on these studies, it was concluded that the efficient transport of CsA results from its ability to reduce its interactions with the aqueous solvent, driving the transport of CsA from the aqueous phase and through the cell membrane. Firstly, this relies on the presence of non-canonical N-methylated amino acids, which reduces hydrogen bond-mediated interactions with the aqueous solvent.^25^ Secondly, conformational flexibility allows CsA to exist in an “open” conformation in aqueous solvents and a “closed” conformation when entering a lipid bilayer, thereby further modifying the hydrogen bonds available for interaction with the solvent.^24^ Finally, the cyclic structure of CsA allows it to bury some of its polar backbone, thus concealing it from water.^21^ Despite these unique features, CsA suffers from low solubility, and therefore the clinically approved product Neoral is formulated as a self-nanoemulsifying drug delivery system (SNEDDS) forming oil-droplets smaller than 150 nm. In addition to facilitating a rapid and uniform drug release, the fatty acid-based excipients in SNEEDS serve as PEs by directly leading to an increase in intestinal permeability as well as inhibiting *p*-glycoprotein efflux and cytochrome P450-3A4 mediated CsA metabolism.

### 2.2 Desmopressin

Oral Desmopressin acetate (DDVAP) is a nonapeptide with a six-amino acid ring structure that has been used for treatment of central diabetes insipidus and primary nocturnal enuresis since the 1980s.^5^ DDVAP is a synthetically-made analog of arginine vasopressin with two modifications, a de-amination of the first amino acid and a substitution of the eighth amino acid replacing l-arginine by d-arginine. Both of these modifications strongly enhance the intestinal stability of DDVAP relative to native arginine vasopressin, mainly by reducing enzymatic degradation. DDVAP has been suggested to transport across the intestinal cell layer by passive permeation, most likely by the paracellular route,^26,27^ however the oral BA of the commercial DDVAP product Minirin is only 0.17% in humans.^5^ Its exceptional potency is the only reason why Minirin remains therapeutically viable at such an extremely low BA.

### 2.3 Oral semaglutide

**Fig. 2:**
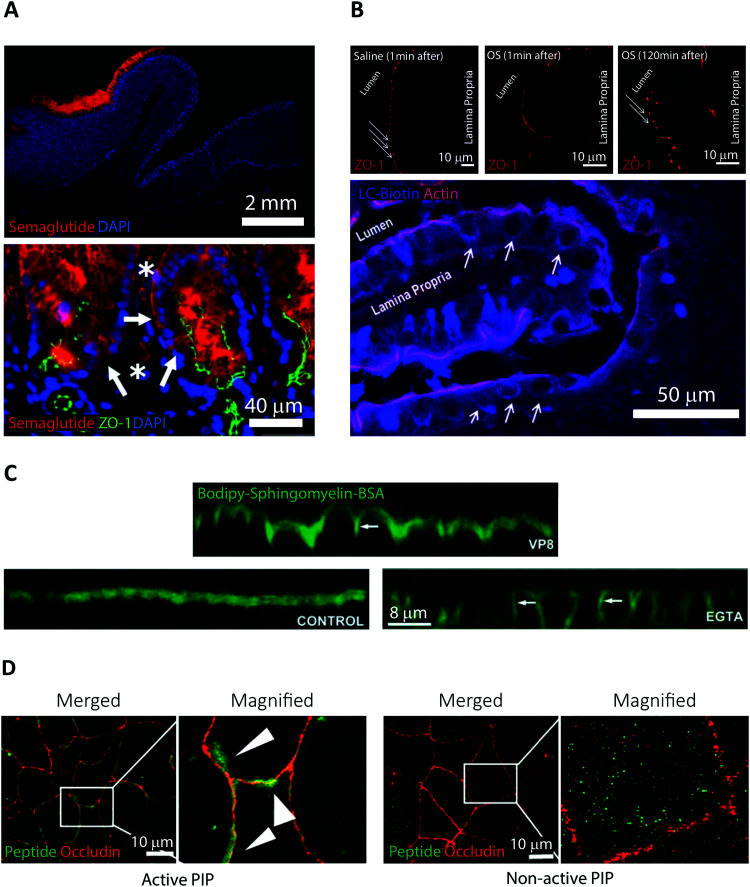
Examples of fluorescence imaging used to elucidate peptide and PE transport mode of action. (A) Immunofluorescence imaging of canine gastric tissue uncovering a transcellular barrier transport mechanism of oral semaglutide. Top, semaglutide (red) and DNA (blue) stains reveal that peptide is predominantly localized to the region in and around of the Rybelsus tablet. Bottom, semaglutide (red) is shown to reside in the cytoplasm of mucosal epithelial cells (white arrows) with intact tight junctions depicted by ZO-1 (green) and DNA (blue). Semaglutide is also detected in capillaries under the epithelium marked by white asterisks. Reproduced from ref. 29 with permission from The American Association for the Advancement of Science, copyright 2018. (B) Immunofluorescence imaging of rat jejunum epithelium reveal that the “Transient Permeation Enhancement” (TPE) delivery technology employed for the octreotide system affects the paracellular permeability. Top, a transient disruption of ZO-1 (red) distribution is induced after one minute incubation with TPE (middle) as compared to saline (left). After 120 minutes incubation with TPE, the ZO-1 organization displays its normal puncta-like morphology (right). Bottom, paracellular flux of the tracer LC-biotin (blue) (white arrows) displayed after incubation with TPE, with the lateral membrane stained for actin (red). Reproduced from ref. 33 with permission from Springer Nature, copyright 2014. (C) Elucidating the ability of the microbial toxin VP8 and the Ca^2+^ chelator EGTA to affect the TJ fence function by imaging caco-2 cell monolayers. In control cells (lower left), imaging of the diffusion marker Bodipy-Sphingomyelin-BSA (green) revealed a staining restricted to the apical cell layer. After addition of either VP8 (top) or EGTA (lower right) clear baso-lateral membrane staining of Bodipy-Sphingomyelin-BSA is evident (see white arrows). Reproduced from ref. 35 with permission from The Company of Biologists Ltd, copyright 2004. (D) Distribution of PIP peptide analogs in caco-2 cell monolayers imaged after 45 min of apical incubation. Binding of Alexa488-streptavidin (green) to active biotinylated PIP peptides (left) or non-active biotinylated PIP peptides (right) reveal a strong colocalization with occluding (red) for active PIP, but a random cytosolic distribution for non-active PIP. Reproduced from ref. 39 with permission from Elsevier, copyright 2018.

Semaglutide is a 31-amino acid linear GLP-1 receptor agonist analog approved under the name Rybelsus in 2019 for treatment of Type 2 diabetes.^5^ Facilitated by the addition of a di-acid C_18_-acylation and substitution of strategic amino acids, semaglutide displays high potency, stability, and long circulating half-life.^21^ These properties compensate for a BA of merely 0.4–1.0%.^28^ However, the large market potential for Type 2 diabetes treatment has resulted in oral semaglutide being described as the most interesting peptide yet considered for oral delivery.^21^ In Rybelsus, semaglutide is co-formulated with the PE salcaprozate sodium (SNAC) and was demonstrated to be exclusive absorbed across the gastric epithelial and thus not in the intestine.^29^ It was shown that SNAC positively affected semaglutide uptake by locally lowering the gastric pH, hereby reducing peptide cleavage by pepsin, and shifting semaglutide towards a monomeric state better suited for transport. *Ex vivo* immunofluorescence imaging on canine gastric tissue was employed for an in-depth analysis of the transport mechanism of semaglutide (Fig. 2A).^29^ An almost exclusive staining for semaglutide around the site of tablet identification strongly supported that close proximity of SNAC and semaglutide were essential for efficient transport (Fig. 2A top). Additional confocal microcopy imaging (see Section 4) revealed intracellular uptake of semaglutide in mucosal cells and staining of the TJ protein ZO-1 confirmed an intact TJ morphology (Fig. 2A bottom). That combined with *in vitro* assays displaying no effect on semaglutide transport upon introduction of TJ modulators like EDTA demonstrates that semaglutide is transported across the gastric epithelium through a transcellular mechanism. This mode of transport concurs with the known function of SNAC as a modulator of the transcellular pathway.^30^

### 2.4 Octreotide

Orally delivered octreotide is a cyclic octapeptide somatostatin-analog that binds with high affinity to somatostatin receptors, hereby blocking the production of growth hormone.^5^ Very recently, octreotide was approved by the FDA (June 2020) for oral treatment of acromegaly under the name Mycappsa.^31^ The Mycappsa delivery system relies on the “Transient Permeation Enhancement” (TPE) technology that solubilizes octreotide in an oily suspension, including the PE sodium caprylate (C_8_).^21^ Despite employing this multi-component delivery platform, the BA of octreotide remains at just 0.5%.^32^*Ex vivo* immunohistochemistry in combination with confocal microscopy were used to investigate the transport mechanisms employed by octreotide in this formulation (Fig. 2B).^33^ Staining rat intestines for ZO-1 revealed a loss of TJ structural integrity within one minute of adding the TPE system and reversal of this effect after 120 minutes (Fig. 2B top). Additionally, using a fluorescently labelled diffusion marker, it was shown that application of the TPE system leads to a paracellular flux (Fig. 2B bottom). This evidence supports that octreotide is transported across the intestinal barrier through paracellular transport *via* a TPE mediated TJ modulating mechanism, induced by C_8_.

## 3. Elucidating permeation enhancers’ modes of action using fluorescence imaging

A recurring obstacle for oral peptide delivery is the poor BAs that are reported in clinical trials to be in the low single digit percentages.^20^ Therefore, there is a quest for discovery, design, and testing of new PEs that could increase the intestinal transport of peptide drugs. Until these endeavors prove successful, oral peptide delivery is restricted to rely on candidates that display elevated intrinsic permeability, high potency, stability, and/or long plasma-half-life. Here, we abstain from giving a comprehensive description of PEs and their use in ongoing clinical trials, since these subjects have been covered extensively in recent, excellent reviews.^5,20,34^ Instead, we highlight some pivotal studies in which fluorescence imaging has been employed in an attempt to elucidate PE modes of action, paving the way for designing smarter and better PEs in the future (Fig. 2).

Traditionally, PEs are divided into classes based on the transport pathway that they affect, mainly paracellular or transcellular (Fig. 1).^20^ However, it is well-established that many PEs affect numerous different pathways simultaneously, often making it hard to pin-point an exact mode of action. In general, paracellular PEs function by disrupting the TJ proteins that ensure a tight barrier between adjacent epithelial cells.^34^ This class is further sub-divided into PEs that either directly affect the TJ proteins or target endogenous cell signaling cascades related to TJ function and integrity. Members of the first sub-group include microbial toxins, which disrupt TJ protein distribution, as shown using fluorescence imaging of *in vitro* cell monolayers.^35,36^ This disruption induces an impairment of the TJ fence function evident from a loss of distinct apical or basolateral membrane staining of lipid reporter systems or membrane proteins (Fig. 2C, VP8). Despite the potent ability of microbial toxins to modulate TJ biology, their clinical use as PEs has remained sparse, mainly due to concerns about toxicity.^21^ The most clinically advanced paracellular PEs are EDTA (ethylenediaminetetraacetic acid) and EGTA (ethylene glycol-bis(β-aminoethyl ether)-*N*,*N*,*N*′,*N*′-tetraacetic acid), which belong to the second sub-group affecting endogenous signaling pathways linked to TJ function.^20^ Both EDTA and EGTA work by chelating extracellular Ca^2+^ ions, causing an efflux of intracellular Ca^2+^ leading to a disruption of TJ integrity. Fluorescence imaging in cell monolayers has shown a similar loss in TJ fence function induced by EGTA as compared to molecular toxins (Fig. 2C, EGTA).^35^ A more directed approach homes in on a specific endogenous pathway, which is believed to reduce toxic off-target effects. One example is the phosphorylation state of the myosin light chain (MLC) complex, which dynamically controls whether the TJ complex is in an “open” or “closed” conformation.^37^ The PIP decapeptide (Permeable Inhibitor of MLC Phosphatase) was developed to specifically prevent dephosphorylation of MLC, keeping the TJ complex in an open confirmation.^38^ This detailed method of action was verified by imaging the intracellular localization of fluorescent PIP peptide in cell monolayers *in vitro*.^39^ The active PIP analog displayed strong spatial colocalization with the TJ complex protein occludin, demonstrating its specific targeting to the site of MLC phosphatase action (Fig. 2D). Single amino acid replacements in control peptides was enough to completely abolish the occludin colocalization observed for native PIP.

Surfactants make up the most abundant group of PEs that potentially affect intestinal transport through the transcellular route.^20^ This group contains fatty acids with intermediate chain lengths (C_8_, C_10_, and C_12_) and acetylated amino acids (SNAC), which are the PEs most abundantly tested in humans.^5^ Originally, these surfactants were believed to facilitate increased transcellular permeability through membrane-insertion-dependent reduction in plasma membrane packing density or through increased peptide hydrophobicity *via* complexation. More detailed method-of-action studies, in which fluorescence imaging has played a central part, have recently questioned the link between surfactants and the transcellular pathway. One example is the C_8_-containing TPE technology (see Section 2.4) that facilitates uptake *via* the paracellular pathway, as shown by fluorescence imaging of rat intestines (Fig. 2B).^33^ Also, a thorough description of the mode of action of C_10_ was recently performed to resolve previous ambiguity of C_10_ function.^30^ The study included *in vitro* cell monolayer imaging of various TJ proteins, showing a clear C_10_ concentration-dependent loss in claudin-5 and occludin localization. Additionally, the authors performed a fluorescence imaging-based high-content analysis, where simultaneous multiplexed detection of fluorescent reporters for nuclear intensity, mitochondrial membrane potential, plasma membrane permeability, and intracellular calcium was achieved at the single-cell level. All evidence suggested that C_10_ increased paracellular permeability *via* a membrane-perturbation induced alteration in intracellular calcium levels, which leads to TJ opening through a MLC regulated mechanism.^30,40^ Additionally, C_10_ has been shown to have a direct effect on membrane fluidity above its critical micelle concentration,^41^ illustrating how PEs, like C_10_, can affect numerous different pathways simultaneously.^21^ The perceived mechanism of the main member of the acetylated amino acid class, SNAC, has also recently been updated. As described in Section 2.3 for the oral semaglutide formulation Rybelsus, SNAC was shown to display formerly unknown buffering and solubilizing effects.^29^ Additionally, SNAC was also shown to facilitate transcellular transport of semaglutide as evident from *ex vivo* fluorescence imaging of canine gastric tissue (Fig. 2A). All the examples provided in this section illustrate how the field is only starting to reach consensus on the PE function, even for PEs extensively used in the clinic. Furthermore, it should be clear that fluorescence imaging is becoming a cornerstone for providing detailed insight, helping to usher in this increased focus on elucidating PE mode of action.

## 4. Fluorescence imaging modalities and single-particle data analysis

### 4.1 Fluorescence imaging modalities appropriate for studying peptide transport

**Fig. 3:**
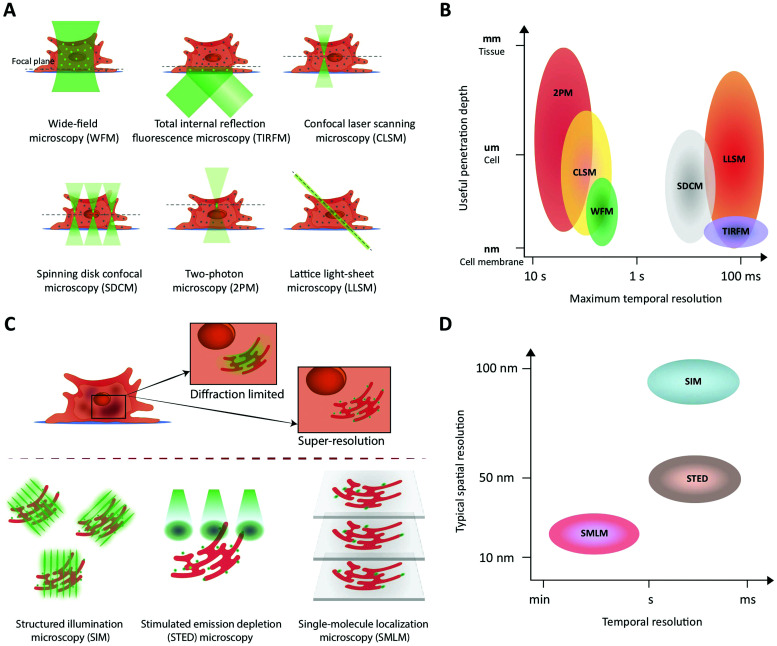
Fluorescence imaging modalities applicable to study peptide transport across membranes and cellular barriers. (A) A schematic of the illumination strategies and the optical sectioning capabilities of different microscopy modalities. The illumination light (green) excites fluorophores in the sample and, effectively, light is collected from a modality-dependent subset of the fluorophores near the focal plane (green dots). (B) A schematic comparing different imaging modalities in terms of their performance with respect to useful imaging depth and maximum temporal resolution when used to image extended samples that are sparsely labeled. In low-light situations, modalities that rely on scanning (CLSM, 2PM) or do not yield efficient background rejection (WFM) are slower than camera-based methods (LLSM, SDCM, TIRFM) that collect light from all pixels in an image plane in parallel. WFM's poor rejection of fluorescence away from the focal plane strongly limits its useful penetration depth due to loss of contrast. (C) A schematic illustrating how the three major classes of super-resolution fluorescence imaging methods overcome the diffraction limit of conventional fluorescence imaging. (D) A schematic comparing the three super-resolution imaging modalities in terms of their typical performance in terms of temporal and lateral spatial resolution.

Fluorescence imaging is widely used in the search for mechanistic insight into peptide transport across membrane- and cellular barriers. It is essential, however, to choose the right imaging modality among the following (ever growing) list of options^17,42^ (Fig. 3):

(i) Wide-field microscopy (WFM) is the standard modality.^17^ It is affordable and consequently broadly available. In wide-field microscopy, the entire sample is exposed to the illumination (bright-field or epi-fluorescence) and imaged with a camera. This mode does not provide resolution along the optical axis. Its achievable contrast is limited by a fluorescent background in samples that extend along the optical axis or when fluorescently tagged molecules of interest, say peptides, are present also in the solution surrounding the sample.

(ii) Total internal reflection fluorescence microscopy (TIRFM) uses an evanescent wave to confine the excitation light to within ∼100 nm of the surface of a coverslip.^43^ This enables single-molecule studies even in extended samples because most of the sample is not illuminated. For the same reason, TIRFM is limited to processes occurring in proximity of the coverslip surface, such as molecular motion in the plasma membrane and early steps of molecular uptake mechanisms.

(iii) Confocal laser scanning microscopy (CLSM) differs fundamentally from the above by scanning the sample with a focused spot of excitation light.^44^ Emitted light is simultaneously collected with a photo detector, but only from the focused illuminated spot on the sample in the focal plane of the objective. All other light is blocked with a screen containing a ‘pinhole’ in the optically conjugate plane. Thus, spatial resolution here originates from the lateral scanning mode of the excitation combined with axial light selection by the pinhole. This also permits optical sectioning, thus providing three-dimensional (3D) spatial resolution in extended samples (∼100 μm). Fast scanning modes are possible, but the modality suffers from high photobleaching rates, since the excitation light is not limited along the optical axis to the part of the sample from which light is collected.

(iv) Spinning disk confocal microscopy (SDCM) essentially parallelizes the confocal illumination and acquisition through multiple pinholes in a rotating disk, relying on a camera to collect the emitted light.^17^ Compared to CLSM this modality has lower photobleaching rates and faster acquisition rates but lower effective penetration depth (∼10 μm), limited by light-collection crosstalk between neighboring pinholes at deeper penetrations.^45^

(v) Two-photon microscopy (2PM) is the preferred modality for deep imaging (∼1 mm) into tissue, model organisms, and on-chip model systems.^42,46^ Here, the fluorescent label is simultaneously excited by two near-infrared photons. The longer excitation wavelength reduces scattering in the sample, while the non-linear multi-photon excitation process strongly confines the excited volume even in scattering samples. This makes pinholes redundant and suppresses the background. The light emitted is collected by a photodetector, which makes collection insensitive to moderate scattering of emission. The major drawback of the modality is its relatively low acquisition speed in practice.

(vi) Lattice light-sheet microscopy (LLSM) now offers prolonged intra-cellular imaging of single molecules. All light-sheet based microscopies uses a second objective to illuminate selectively a plane of interest in the sample.^47–49^ In conjunction with a camera, these modalities allow imaging of an entire plane during each exposure. Recently, the lattice light-sheet microscope was developed to illuminate the sample with an ultra-thin light sheet. This effectively achieves extremely efficient background rejection in selected planes of living cells and optically transparent organisms.^50–52^ This modality was recently combined with adaptive optics that permit correction for optical distortions created by the sample itself, which allows for deeper imaging (∼100–200 μm) into tissues by tiling independently-acquired fields of view.

**Fig. 4:**
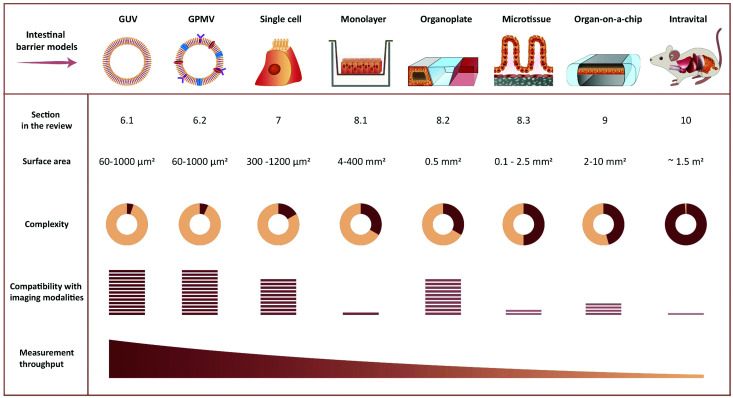
Representative illustration of imaging-compatible biological models applicable to study peptide transport across the intestinal barrier. The pros and cons of various model systems are qualitatively compared with respect to complexity, compatibility with imaging modalities, and measurement throughput.

The choice of experiment, imaging modality,^17^ and image analysis depends, obviously, on the scientific question at hand. This choice subsequently defines the resolution with which one can provide answers. Light scattering and background in thick samples, for example, are factors that determine the level of detail that can be imaged. Also, the working distance of the objective is an important parameter when imaging peptide transport across various models of the intestine. The physical dimensions of the model of interest (Fig. 4) determine the range of applicable objectives. The need for long working distance objectives to image traditional intestinal barrier models (see Sections 8 and 9) often prevents high-resolution imaging.^53^ Oil-immersion objectives provide the highest numerical apertures (NAs), and hence the highest resolution, but typically have a working distance below 130 μm.^54,55^ This leaves an effective depth of imaging around 100 μm in typical imaging conditions. Water-immersion objectives provide NAs up to 1.3 but offer a much larger range of working distances, up to 500 μm. If a cover glass can be omitted, water dipping objectives can provide very long working distances for high NAs (for example 60×/1.0 NA with 2.0 mm working distance). In all cases, the working distance increases if one can compromise on NA, magnification, and need for optical corrections. The choice of objective in turn determines the microscope modality and the magnification that can be used. Importantly, the temporal resolution in all modalities is either limited by the scanning speed of the microscope or the acquisition rate of the camera. When light is abundant, *e.g.* when imaging biological structures associated with bright or multiple fluorescent labels, the limited speed of the hardware limits time resolution. When light is scarce, *e.g.* when imaging single (peptide) molecules or structures labeled with a single or a few fluorophores, the rate at which photons are detected instead limits time resolution. In the latter case, recording with a camera (imaging sensor) in general yields better temporal resolution, provided that background fluorescence can be efficiently rejected (TIRFM, SDCM, LLSM), than scanning with a point detector (CLSM, 2PM), since all pixels in the image are acquired simultaneously and photons are usually collected with a higher quantum efficiency (Fig. 3B).

Phototoxicity is an important potential artifact, when using imaging modalities to study peptide transport in a live cell setup.^56^ The excitation light used to illuminate the cells will react with naturally occurring compounds like flavin and porphyrin inside the cells.^57^ These can then be degraded in a process creating reactive oxygen species that are detrimental to cell health and thus effect the experimental outcome. Reactive oxygen species can also be created when fluorophores undergo photobleaching. Some steps in the experimental setup can be taken to reduce phototoxicity: the detection scheme can be optimized to lower the effective illumination dose of the sample.^58^ Additionally, modification of the buffer solution by adding antioxidants to scavenge reactive oxygen species or removing certain vitamins to reduce photobleaching have been demonstrated to reduce overall phototoxicity. Such specialized buffers for live cell imaging are commercially available.^57^ However the most efficient way of reducing phototoxicity is to selectively only illuminate the currently imaged volume and not the entire depth of the sample. Selective illumination is a cornerstone in the emerging imaging methods TIRFM, 2PM and LLSM (Fig. 3A). Especially LLSM offers the possibility of only illuminating the focal plane, which is then scanned rapidly through the cell sample, allowing for time-dependent 3D imaging, often termed four-dimensional (4D) microscopy, with greatly reduced phototoxicity.^51^

### 4.2 How single-particle/molecule data analysis may be used to gain mechanistic insight on peptide transport

One path to mechanistic insight using fluorescence microscopy focuses on the detection and tracking of single molecules and/or particles. Tracking may enable quantification of heterogeneities in uptake pathways for individual particles. Heterogeneities may, in turn, identify the roadblocks in cases where transport of peptide drugs across a barrier is not complete, for example due to (partial) sequestering in endocytic pathways. The use of single-particle tracking in biophysical and pharmaceutical research has been reviewed on several occasions.^59–64^ In the context of peptide translocation, single-particle tracking has predominantly been applied to study cell-penetrating peptides, both in artificial model membranes^65^ (see also Section 6.1) and in live cells^66–68^ (see also Section 7). In this way, the transport mechanisms of either the peptide itself or of its model delivery system have been elucidated.

Observation and analysis of the internalization, transport, and fate of individual peptides are desired to study uptake heterogeneities at the molecular level. Dynamic imaging of individual peptides is limited by only a single fluorescent molecule being present on each peptide (see Section 5). Despite continued progress in fluorophore development,^69^ it remains a challenge to record a sufficient number of photons from the peptide with sufficient temporal resolution to follow its whereabouts. Single peptides diffuse fast, which results in motion blur during exposures long enough to record a supposedly sufficient number of photons. If stacks along the optical axis are required for full 4D resolution, the challenge is even bigger. On the other hand, a crowded cellular environment or interaction with the cell membrane or various organelles slows diffusion. Thus, single-peptide studies in such environments have their temporal extent limited by the fluorophore stability rather than by the acquisition speed of the microscope.

In case of sufficiently low (labeled) peptide density, LLSM allows 4D tracking of individual molecules/peptides in live-cells for extended durations. This modality should be particularly suited to study individual peptides in direct translocation across the membrane,^70–72^ a process that is difficult to capture due to the limited number of photons available. On the other hand, endocytic uptake of multiple individual peptides or aggregates results in a high peptide density in the endosomes. This makes more photons available, as long as the fluorescent peptide remains in the compartment,^73^ which enables single-particle (endosome) resolution in 4D using SDCM.

Successful analysis of single-particle/molecule tracking data relies on three main steps:

(i) Detection of spots and their linking into trajectories. This may be considered a precursor step to a single-particle/molecule analysis.^60,74–77^

(ii) Sub-pixel resolution location of the fluorophore(s) that caused the detected spot trajectories. Typically, this localization is done by fitting a 2D or 3D model for a spot's intensity distribution to the measured spots. The precision of the fluorophore's location that results from this localization analysis depends critically on the number of photons in the measured spot.^78,79^ A plethora of packages for automated 2D and 3D localization analysis exist, and a large fraction of them have had their performances compared across a number of data sets.^80,81^

(iii) Characterization of the underlying motion at the single-object level based on the high-precision trajectories obtained.^59,60,82^ Most approaches rely on the mean-squared displacement of particle trajectories, but simpler, more rigorous alternatives exist for particles that exhibit normal diffusion.^83,84^

In all steps, the automated analyses have obvious advantages in terms of ease-of-use. Unfortunately, one-size-fits-all tools may result in suboptimal localization analyses.^85^ Care should be taken throughout, since choices made by the user in every step may affect conclusions.

Single-particle/molecule tracking is often conducted as co-localization studies, which allows real-time tracking of multiple objects. This may be quantified as correlations, *i.e.* synchronized motion, between spectrally separated images of a drug/peptide and any labeled cellular entity of interest.^86,87^ More detailed information is attainable by accurately correlating color channels over time in an experiment to provide relative positions of drug/peptide and carrier compartment, not just information on their colocalization.^88^ Relative positions can potentially discriminate between the peptide being transported on the inside or the outside of a membrane of a compartment to further elucidate transport mechanisms after successful uptake.

Highly supervised data analysis is under pressure from the increased use of high-resolution fluorescence imaging. The large amounts of data produced cause a demand for automated analyses. Thus, machine learning strategies^89^ towards analysis currently proliferate in many branches of science. In single-molecule based localization microscopy, such algorithms may well be used to automate and speed up analyses^90^ when one knows what the machine should learn to look for, *e.g.* specific biological structures and/or dynamics.^89,91^ However, in the exploratory phases of data analysis and improvements of experimental designs, their use seems limited.

### 4.3 Super-resolution fluorescence microscopy

The microscopy modalities described above are all limited by diffraction to a spatial resolution of a few hundred nanometers. Consequently, labelled structures and/or molecules that are separated by less than this distance cannot be discriminated in images, which occludes the nanoscale organization of biological structures. In the past two decades, however, this fundamental resolution limit has been surpassed by various super-resolution methods for optical microscopy. These methods have had a major impact on the visualization and quantification of biological structures and processes at the nanoscale, and, as a result, they have been reviewed on many occasions.^92–96^ We refer the reader to these excellent reviews for a detailed account of the methods and their usages in various contexts. Here, we provide a brief overview of the classes of methods and highlight their strengths and limitations in the context of cellular transport. In this light, it is important to realize that these methods originally were conceived as tools to circumvent the diffraction limit in the imaging of structures, but more recent developments of the methods, however, also permit their use to probe dynamics. The different requirements of those two applications are important to be aware of when choosing a super-resolution fluorescence modality for an application. With that in mind, super-resolution microscopy has been applied to study many structures and processes that are relevant in the context of cellular transport. Examples are: the nanoscale architecture and dynamics of cellular organelles, such as endosomes, the heterogeneity and mobility of cell-membrane associated proteins, intra-cellular motion of proteins, and dynamics of internalization and cellular trafficking of nanoparticles.^92–98^

In general, super-resolution fluorescence methods can be divided into two main classes. The first class uses engineered illumination of the sample to circumvent the diffraction limit. The second class consists of various single-molecule localization-based methods, in which fluorophores are separated in space and/or time and then localized with nanometer resolution, using tools identical to those described above for single-molecule tracking (see Section 4.2).

(i) Structured illumination microscopy (SIM)^99,100^ falls in the first class of methods. It exposes the sample to multiple high-spatial-frequency illumination structures, typically parallel lines that are phase-shifted and rotated relative to each other (Fig. 3C). This encodes sub-diffraction-limited features from the sample in the resulting images. A super-resolved image of the sample may then be obtained by deconvolution of the images. In its simplest implementation,^99,100^ SIM essentially combines two diffraction-limited sources of information. Consequently, it results only in a doubling of resolution relative to conventional diffraction-limited imaging (Fig. 3D). SIM allows straightforward multiplexing with different colors of fluorophores, is compatible with live-cell imaging, since it does not require high illumination intensities, and does not require complicated sample preparation. The temporal resolution is in the millisecond-to-second range, limited by the number of structured illumination patterns necessary for reconstruction/deconvolution (Fig. 3D).

(ii) In stimulated emission depletion (STED) microscopy,^101,102^ a super-resolution image is obtained by scanning the sample with an effectively sub-diffraction limited excitation spot. To this end, the conventional confocal excitation spot is scanned synchronously with a second, doughnut-shaped depletion spot (Fig. 3C). The latter beam depletes excited fluorophores before they decay to the ground state by emission of fluorescence. Sub-diffraction-limited resolution is achieved through the non-linear dependence of STED on intensity of the depletion light: Intensities above a certain threshold deplete all fluorophores. Thus, only fluorophores positioned in the middle of the “hole” in the doughnut will avoid depletion and hence emit fluorescence. Consequently, the spatial resolution of STED is determined by the sharpness of the doughnut around its minimum. Typically, a lateral resolution of ∼50 nm is achieved (Fig. 3D). Fluorophores should be chosen so they are compatible with both lasers of the STED setup. The large intensity required for the depletion laser may result in phototoxicity to the sample, which may hinder prolonged biological imaging with this modality. The temporal resolution is in the millisecond to second range and is limited by the need to scan the entire field of view (Fig. 3D).

(iii) Single-molecule localization microscopy (SMLM) is a class of methods that achieve sub-diffraction-limited resolution by precise localization of single fluorescent probes in a sample. To do this, the sample is imaged repeatedly, with only a sparse subset of fluorophores activated in each frame (Fig. 3C). Popular methods include (direct) stochastic optical reconstruction microscopy (STORM,^103,104^ dSTORM^105^), photo-activated localization microscopy (PALM^106^), and point accumulation for imaging in nanoscale tomography (PAINT^107^), which primarily differ in the means by which they create the sparse subset of active fluorophores. In a given image, each active fluorophore is localized with a precision that is limited, in principle, only by the number of photons observed from it (see above). In practice, however, other factors, *e.g.* labelling density and sample stability, also influence the resolution. A final resolution of ∼20 nm is not uncommon for biological samples (Fig. 3D). The temporal resolution is limited by the number of images required for sufficient coverage of the targeted structure, which typically takes seconds to minutes to acquire (Fig. 3D). Fluorophores must be chosen to be photo-switchable or have appropriate blinking dynamics in order to be compatible with the super-resolution method or, in the case of PAINT, be conjugated to molecules with appropriate binding kinetics relative to the structure of interest. All methods are compatible with various field-wide illumination schemes, such as WFM, TIRFM, and (L)LSM (see Section 4.1).

For studies of single-particle and single-molecule dynamics in live cells, both STED and the various SMLM methods may be combined with single-particle tracking (see Section 4.2) and dual-color labeling strategies for co-localization.^97,98,108^ Due to its scanning nature, STED may achieve sufficiently high temporal resolution by compromising on the size of the field of view. On the other hand, SMLM methods, such as sptPALM (single-particle tracking PALM), simultaneously solve the two problems of sufficiently sparse labeling and replenishing of labels for imaging, since they only view a subset of the fluorescent molecules at the same time. This increases throughput by orders of magnitudes without any additional sample preparation steps. In this mode, SMLM is not limited by the number of images required for imaging of a structure, since only individual molecules are tracked through consecutive frames, until this tracking is repeated for another subset of molecules.

Recent methodological developments in microscopy have yielded a series of methods that essentially are hybrids of the different modalities described above. Notably, MINFLUX^109,110^ uses multiple exposures of a doughnut-shaped illumination beam and the relative number of photons observed from a fluorophore to localize and track it. For given resolution, this requires an order of magnitude fewer photons than conventional single-molecule tracking, which enables MINFLUX to track individual molecules with unprecedented temporal resolution. Its scanning configuration, however, limits throughput. In its wake, a series of methods have emerged, which do not suffer from limited throughput. They use wide-field illumination structures (similar to those used in SIM) and thereby double the resolution of localization of individual molecules in 2D compared to what is achieved with uniform illumination.^111–113^ Very recently, similar methods have been developed for improved axial localization using illumination structured along that dimension.^114,115^ These wide-field methods are yet to be applied to imaging dynamics, however.

## 5. Fluorescent labeling and biophysical characterization of peptides

### 5.1 Potential artifacts introduced to peptide properties and behavior by fluorescent labeling

**Fig. 5:**
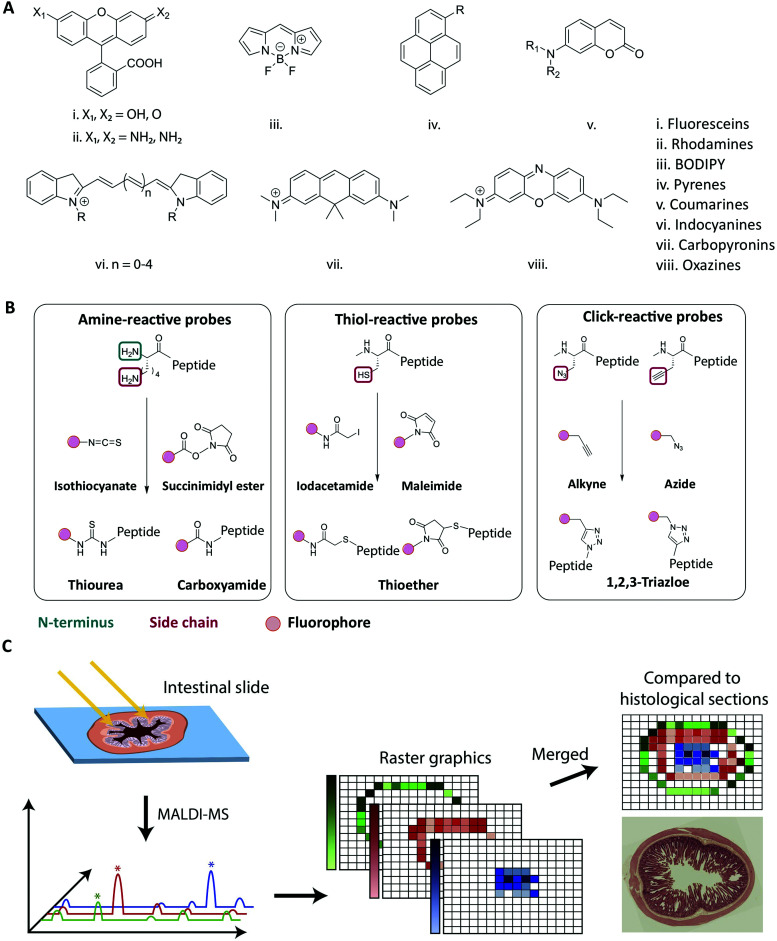
Strategies for fluorescent labeling of peptides. (A) The chemical core structures of the most common and commercially available fluorophores. The properties of the fluorophore derivatives can be chemically tailored, by changing the substitution pattern of the respective core structures or by the addition of chemical moieties. This results in a great variety of different fluorophores available for many different applications. (B) The chemistry behind the commonly employed fluorophore conjugation techniques using either amine-, thiol- and click-reactive probes. Amine-reactive probes target amine groups like lysine residues or the N-terminus. A wide variety of different amine-reactive probes is available. This makes it an advantageous method, when labeling peptides during SPPS. Thiol-selective probes are advantageous, when labelling peptides in solution. Due to the low abundance of cysteine residues this conjugation techniques results in a high regioselectivity. Click-reactive probes offer the advantage of bioorthogonality, however, an unnatural amino acid with an alkyne or azide moiety needs to be incorporated into the peptide sequence. (C) Schematic overview of the MALDI-MSI method for the evaluation of peptide degradation or modification *in vivo*. A frozen intestinal section is first cryosliced and placed on a glass slide before a matrix is applied and the MALDI-MS is performed. For each pixel a mass spectrum is obtained allowing the creation of raster graphics which can be compared to histological sections. Reproduced from ref. 181 with permission from American Chemical Society, copyright 2021.

Fluorescence-based imaging studies of peptide transport across membranes and cellular barriers require the creation of a peptide–fluorophore construct.^116–120^ Creating such constructs potentially changes the physiochemical properties of the peptide, which might affect the transport behavior as compared to the unlabeled peptide. Both the choice of fluorophore and conjugation strategy has been shown to influence the properties of the labeled peptide (Fig. 5).^121–124^ Fluorophore size and hydrophobicity/hydrophilicity may influence the solubility, aggregation properties and partitioning coefficient of the labeled peptide (Fig. 5A).^121^ The conjugation strategy such as the labeling position and conjugation chemistry may further impact amphipathicity and secondary structure of the labeled peptide (Fig. 5B).^122^ A direct experimental artifact reported for some fluorescently labeled peptides is fluorophore mediated non-specific binding of peptides to glass surfaces or other substrates during microscopy, which can hamper single-molecule tracking and cause inaccuracies when using such setups to track peptide-membrane interactions.^125^ It has been shown that the extent of fluorophore hydrophobicity (Fig. 5A) highly influences the extent of unspecific binding and caution should be exercised when choosing the respective fluorophore.^124–126^ When employing fluorescently labeled peptides for studying transport across cellular barriers it is important to acknowledge that the fluorophore label has been shown to affect both the initial membrane interaction as well as the intracellular trafficking of peptides.^121,122,124^ Based on a comprehensive screen of a wide range of commonly used fluorophores it was concluded that the fluorophore:membrane interaction propensity varied greatly. Thus, researchers should always strive to select fluorophores known to not drive the membrane interaction on its own. Once the peptide is taken up by the cell, the fluorophore can also influence the intracellular distribution of the conjugated peptides.^121^ Depending on the size and shape of the fluorophore, different localization patterns were observed for the same peptide, demonstrating how fluorophore labeling can bias the interpretation of intracellular transport data.

In addition to phototoxicity during imaging, it has also been shown that the mere presence of the fluorophore conjugate can lead to an increased cellular toxicity.^121^ A possible mechanism is a loss of membrane integrity due to the fluorophore mediated enhancement of peptide-membrane interactions supported by the physiochemical properties of the fluorophores having a direct influence on the extent of the cytotoxicity. Screening a selection of fluorophores determined that neutral hydrophobic fluorophores or negatively charged fluorophores conferred less cytotoxicity as compared positively charged, hydrophobic fluorophores.^121^

### 5.2 Strategies for fluorescent labeling of peptides

As outlined in the previous section, it is essential to understand the impact of the fluorophore on both the transport across membranes as well as the intracellular trafficking. This impact should, if at all possible, be evaluated in the context of the native peptide by performing a structure/activity analysis and mechanistic transport study. However for studies where fluorescence imaging is the sole experimental platform, any potentially detrimental effect of fluorophore conjugation can be delineated by comparing results from identical peptides labelled with chemically distinct fluorophores. Other factors for the choice of fluorophore depend on the experimental design, including the density of fluorescently-labeled peptides under study (see Section 4.1), and should be made based on its spectroscopic properties, including absorption and emission spectra, the molar extinction coefficient, quantum yield, Stokes shift, and its propensity for quenching and bleaching.^127^

Traditionally, peptides are either produced by recombinant expression or synthesized using solid-phase peptide synthesis (SPPS).^128,129^ The synthetic approach allows for full design-flexibility over the peptide sequence, enabling the introduction of chemically reactive handles available for bio-conjugation, such as the labeling with fluorophores. The conjugation of fluorophores to peptides^130,131^ can be achieved by modifying the isolated peptide in solution, by adding the fluorescent label to side chain-protected polymer-bound peptides during SPPS^132,133^ or by incorporating pre-labeled amino acids into the sequence.^134,135^ When introduced in solution, the applied conjugation chemistry should be efficient, regio- and chemoselective, to ensure the formation of the desired product in high yield. When introduced during SPPS, the fluorophore should furthermore be compatible with deprotection- and cleavage conditions, as well as heating, if applied. In Fig. 5A the chemical core structures of the most commonly used fluorophores are depicted. By adding or changing the substitution pattern of functional groups, the properties of the respective fluorophores can be tailored. The most common commercially available fluorophores are derivatives of the shown core structures. For example, the widely used fluorophores fluorescein isothiocyanate (FITC) and carboxyfluorescein (CF) are built around the fluorescein core structure (i in Fig. 5A). The frequently used fluorophores Alexa Fluor 488, Atto 488 and 5-carboxytetramethylrhodamine (TAMRA) are all built around the rhodamine core structure (ii in Fig. 5A.). Bodipy and a number of derivates is designed around the main Bodipy structure (iii in Fig. 5A). Alexa Fluor 405 is built around the pyrene core structure (iv in Fig. 5A) and Alexa Fluor 350 or 430 are based on the coumarine core structure (v in Fig. 5A). The indocyanine core structure can be found in *e.g.* Cy5 or Alexa Fluor 647 (vi in Fig. 5A), Atto 610 builds around carbopyronin (vii in Fig. 5A) and Atto 655 represents a derivate of oxazines (viii in Fig. 5A). Many fluorophores, for example TAMRA and CF, contain reactive functional groups such as hydroxyl or amine groups. Consequently, the fluorophore preferably should be introduced in the final step, in order to avoid side-reactions during SPPS.^132,136^ In cases where the fluorophore is in short supply due to cost or challenges in synthesis, the solution phase conjugation is a stoichiometric, cost-effective alternative to the SPPS approach, in which a larger excess of reagents is traditionally used.^137^

The most commonly used fluorophore conjugation chemistries utilize amine- and thiol-reactive probes and the alkyne/azide functionalized probes (Fig. 5B). The amine-reactive fluorophores are mainly acylating reagents such as activated esters^138^ or isothiocyanates^139^ (Fig. 5B, left). When they react with a peptidic amine, such as the N-terminus or a sidechain amino group, an amide bond or a thiourea will form. Although thioureas are less stable than amide bonds, isothiocyanates such as FITC and tetramethylrhodamine isothiocyanate (TRITC) are relatively cheap and thus are still widely used.^136,137,140^ The thiol-reactive fluorophores are mainly alkylating reagents, such as iodoacetamides^141^ or maleimides^142^ (Fig. 5B, middle). The thiol-reactive fluorophores react with free cysteine residues forming a thioether bond, and the relatively low abundance of cysteine in in peptides makes regioselective fluorescent labeling possible. The majority of fluorescent dyes are commercially available as amine- and thiol-reactive probes. In an alternative bio-conjugation approach, alkyne- or azide-functionalized probes and peptides are used (Fig. 5B, right).^143,144^ In the presence of a copper(i) catalyst, alkynes react with azides forming a very stable 1,4-substituted 1,2,3-triazole, often referred to as a “click” reaction.^145,146^ It is biorthogonal, meaning that the reactants’ chemical handles possess a unique reactivity that is orthogonal to naturally occurring functional groups.^144,147^ The azide/alkyne moiety can be introduced into peptides by incorporating unnatural amino acids, either into the peptide sequence during synthesis^148^ or by post-synthesis bio-conjugation of an azido/alkyne moiety.^149^

The broad range of commercially available fluorophores and their straightforward bio-conjugation and synthesis provide a high degree of flexibility in the peptide–fluorophore design. However, it is imperative to ensure that both the spectroscopic performance of the attached fluorescent dye and the basic functional properties of the peptide are minimally disturbed by the labeling process. Alternatively, any change should be characterized and understood. The next section details how to evaluate the effect fluorescent labeling infer on peptide properties and behavior and we outline the additional possibilities for peptide characterization offered by the fluorescent label, as well as the potential pitfalls associated with their use.

### 5.3 Basic characterization of fluorescently labeled peptides

While fluorescent labeling of peptides may give insight to the mechanistic pathway as they translocate across barriers (Fig. 1, right), the fluorescent labeling may directly affect a peptide's solubility,^150^ conformational dynamics,^151^ oligomerization and fibrillation behavior,^152–154^ membrane interactions,^122^ and receptor binding.^155^ Before studying fluorescently labeled peptides in complex biological environments, it is thus important to evaluate the physicochemical properties of the peptides and how they compare to the unlabeled peptide.

In principle, fluorescently labeled peptides may be evaluated using common peptide characterization techniques,^156^ including dynamic light scattering, size-exclusion chromatography with multi-angle light scattering, ultracentrifugation, circular dichroism, nuclear magnetic resonance, and fluorescence from extrinsic dyes (*e.g.* thioflavin T). However, for several of these techniques, there is a risk that the fluorophore attached to the peptide might disturb the measurement. For example, in assays with extrinsic fluorescent dyes, the peptide-attached fluorophore may obscure the signal of interest.^122^ Likewise, the presence of a fluorophore may reduce the sensitivity and accuracy of light scattering-based techniques.^157^ This calls for careful choice of the techniques used for characterization of fluorescently labeled peptides and execution in a manner compatible with the fluorophore.

Fluorescent labeling of peptides, however, also opens new opportunities for studying peptide properties, for instance investigation of the peptides with fluorescence fluctuation-based techniques.^158,159^ An important example of this class of techniques is fluorescence correlation spectroscopy (FCS).^160^ In FCS, the emission intensity of fluorescent molecules diffusing in and out of a small confocal detection volume is recorded, and the temporal fluctuations of the intensity are analyzed to obtain information about the concentration and diffusion properties of the molecules. FCS may thus report on the oligomerization and aggregation of fluorescently labeled peptides, since multimeric peptide species have a smaller diffusion coefficient than monomeric peptides.^161^ Similarly, FCS may reveal conformation changes of fluorescently labeled peptides in cases where these changes are associated with an altered diffusivity of the peptides.^162^

Fluorescent labeling may also provide the opportunity of studying peptides using Förster/fluorescence resonance energy transfer (FRET).^163^ This non-radiant energy transfer process may take place between two fluorophores when the emission spectrum of the one fluorophore (the donor) overlaps with the excitation spectrum of the other fluorophore (the acceptor). The efficiency of the process depends on the spatial proximity and relative orientation of the donor and acceptor, and therefore, FRET may provide information about the relative nanometer scale distance of the two fluorophores. In samples with mixtures of peptides labeled with either a donor or an acceptor, it is possible to use this information to study peptide oligomerization and determine the stoichiometry of peptide complexes.^164,165^ Additionally, if a given peptide is labeled with both a donor and an acceptor, it is possible to use the information to investigate the conformational state and dynamics of the peptide.^166^

Any research using fluorescently labeled peptides assumes that the fluorescence signal corresponds in space and time to the peptide of interest. Obviously, this is true only if the peptide–fluorophore construct is not degraded. Ensuring the absence of degradation is especially important in the design and study of peptide transport across the intestinal barrier, as the intestine is a particularly harsh environment.^7,8,167^ The biochemical barrier is constituted by a range of peptide degrading enzymes including pancreatic proteases in the intestinal fluids, brush-border membrane peptidases at the cellular interface, and intracellular enzymes within the enterocytes.^168–170^ Peptide degradation will in most cases not affect the fluorescent label. Consequently, the peptide's stability has to be assessed independently of the attached fluorophore. To this end, three alternative avenues are pursued: either (i) the peptide is incubated *in vitro*, simulating the intestinal environment; or (ii) the peptide is administrated *in vivo* and subsequently studied *ex vivo*, *e.g.*, by extracting blood samples; or (iii) the peptides are studied directly in the native intestinal environment. In option (i), researchers attempt to recreate the intestinal environment *in vitro* by incubating peptides in chemically simulated intestinal fluids (SIFs) that contain the naturally occurring pancreatic enzyme mixtures, bile acids, and phospholipids as well as having the relevant pH.^171,172^ Another possibility is to incubate peptides in human aspirates from the upper gastro-intestinal tract, which mimics the *in vivo* environment closer but requires advanced sampling from human subjects.^173–175^ Option (ii) exposes the peptide to the native intestinal environment prior to *ex vivo* analysis,^176,177^ traditionally using the same analytical chemistry techniques as employed for studying peptides in option (i). These techniques most often include high-performance liquid chromatography- and/or mass-spectrometry-based analysis methods, which offer insight on peptide stability, potential chemical modifications as well as degradation kinetics.^178–180^

Options (i) and (ii) can indicate if the peptide of interest is still intact when exposing it to the environment of the intestine. However, they neglect the possibility of observing location dependent degradation or modification patterns (*e.g.* during transcytosis). For option (iii), techniques like matrix-assisted laser desorption/ionization mass-spectrometric imaging (MALDI-MSI), allows for visualization of the stability and distribution of peptides directly in the tissue, *e.g.*, across the small intestine.^181^ This technique has been employed to elucidate how di-peptides were partially degraded by brush-border enzymes, providing an explanation for low *in vivo* absorptions (Fig. 5C).^182–184^ The combination of MALDI-MSI and fluorescence imaging provides a powerful combination to verify the integrity of the peptide of interest but also opens up possibilities to learn about spatial degradation and modification mechanisms during peptide transport across the intestinal barrier.

Of relevance, the fluorescence-based techniques mentioned in Section 4 generally have in common that they allow for evaluation of the peptides not only in simple aqueous solutions but also in complex biological environments. Accordingly, they may be applicable for studying the behavior of peptides in the various experimental setups described in the following sections.

## 6. Membrane model systems

The cellular interaction of peptides starts at the level of the plasma membrane. The first step of deciphering the intestinal transport mechanisms of peptides is thus to understand their membrane interactions. Imaging-compatible membrane model systems represent a useful tool for shedding light on these interactions.

### 6.1 Image-based peptide–membrane interaction and translocation studies using artificial membrane models

The simplest approach to studying peptide–membrane interactions is to use artificial membrane models. To obtain free-standing artificial membranes compatible with imaging, giant unilamellar vesicles (GUVs) are often used (Fig. 4, GUV).^185–189^ These vesicles are usually either formed by electroformation or by spontaneous swelling to be >5 μm in diameter, approximately the size of mammalian cells.^190^ To ensure their biological relevance, the vesicles are typically prepared to consist of unsaturated phosphatidylcholines,^187–189^ which is the most abundant type of phospholipid in mammalian plasma membranes.^191^ Sometimes, they are also prepared with other lipid constituents to confer additional specific properties on the vesicles, for example, cholesterol to increase membrane rigidity^187^ or phosphatidylglycerol to decrease membrane surface charge.^186,188,192^

**Fig. 6:**
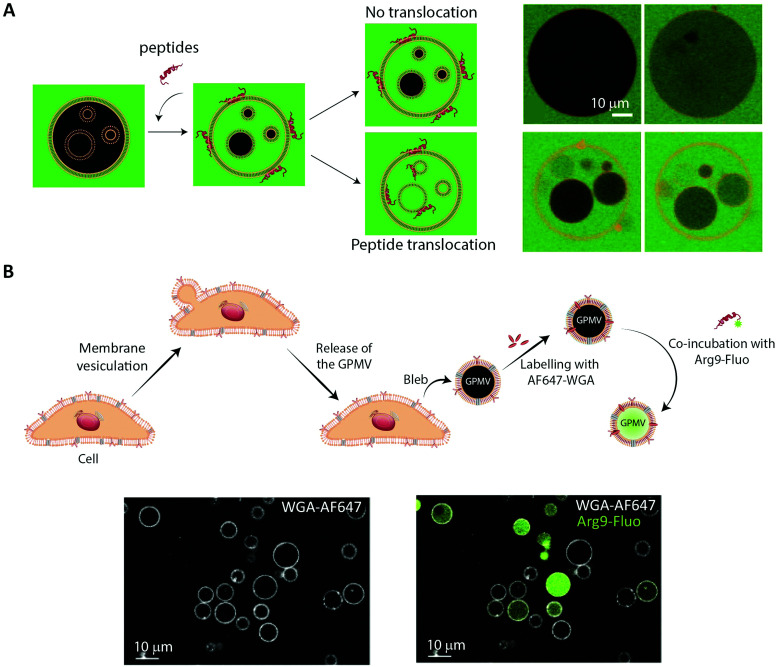
Using membrane model systems to study peptide translocation. (A) Left, schematic illustration showing the concept of the peptide:membrane interaction and translocation experiment using the simplest membrane model system, GUVs. Influx of CF (green) in the inner GUVs constitutes a sensitive method for proving the ability of peptides to translocate across membranes. Right, studying the membrane translocation of fluorescent Transportan 10 analogs (Rhodamine-TP10W, red) using a microscopy-based multivesicular vesicle assay, employing CF influx into GUVs as a function of time (8.5, 14, 33, and 75 min). Reproduced from ref. 189 with permission from American Chemical Society, copyright 2013. (B) Top, schematic illustration of cell blebbing, GPMV formation and peptide transport experiment. Bottom, membrane translocation of the fluorescently labeled nona-arginine (Arg9, green) after 1 h, studied using GPMVs labeled with wheat germ agglutinin conjugated to Alexa Fluor 647 (WGA-AF64, white). Reproduced from ref. 204 with permission from Elsevier, copyright 2016.

GUVs are commonly used to study membrane binding and translocation of fluorescently labeled peptides. The translocation studies are frequently done with GUVs with enclosed inner vesicles, exploiting that peptides can only bind to an inner vesicle if they have translocated across the outer membrane of the GUV (Fig. 6A).^186,188,189^ By this principle, it is possible to determine the kinetics of peptide translocation across the outer membranes if the association to and dissociation from the membranes are not rate limiting.^189^ Furthermore, by comparing the time course of the translocation process to the transport of non-permeable fluorophores across the outer membranes, it is possible to investigate whether or not the translocation process is coupled with membrane permeabilization.^185,187,189^

### 6.2 Using cell-derived giant plasma membrane vesicles for studying peptide membrane binding and translocation

While GUVs have been a steady work horse in the field of peptide translocation studies, they represent a completely artificial membrane system. Thus, their lipid composition are much simpler than that of a cell membrane, and they do not contain any of the membrane proteins making up roughly half the plasma membrane area in cells.^193^ Thus, for more realistic quantitative biophysical peptide–membrane interaction studies, researchers have turned towards more native-like environments than classical synthetic liposomes.^194–196^ Specifically, in recent years giant plasma membrane vesicles (GPMVs) have become increasingly popular (Fig. 4, GPMV). GPMVs are micron-sized single-bilayer structures, created by treating cells with chemicals that induce a controlled blebbing of the plasma membrane (Fig. 6B).^196^ Initially GPMV production protocols relied on mixtures of the protein and lipid cross-linking agent formaldehyde and the reducing agent dithiothreitol (DTT) to weaken the interaction between the cytoskeleton and the plasma membrane followed by volume expansion driven by intracellular pressure.^197^ Although much lower concentrations of formaldehyde is used than for regular cell fixation, this method was still believed to produce less than ideal mimics of the plasma membrane environment.^198^ Less harmful protocols have been developed, including the use of *N*-ethylmaleimide, which do not directly cross-link protein or reduce disulfide bridges.^196^ Still, one needs to be aware that GPMV preparation processes have been reported to impose a lack of actin cytoskeleton, phosphorylated lipids, and strict lipid asymmetry between bilayer leaflets. The key advantage is that GPMVs maintain the diverse lipid and protein composition of the original cellular membrane.^199^ This means that the GPMVs harbor a lipid complexity, which cannot be produced in purely artificial systems, and which can be modified by the choice of initial cell line.^197^ Additionally, cell lines can be genetically modified to express fluorescently labelled plasma membrane proteins, giving the opportunity to use imaging modalities to track peptide:protein interactions in the produced GPMVs. Known regulators of peptide:cell interactions are the glycoproteins and glycolipids making up the pericellular matrix, known as the glycocalyx. Since GPMVs have been shown to retain the glycocalyx after isolation,^200^ they offer the possibility to study how it affects peptide:membrane interactions in a controlled environment. Although GPMVs cannot be used to study the endocytic pathways of peptide uptake, they do represent a well-suited system for elucidating peptide–membrane interaction and direct translocation mechanisms. Thus GPMVs are believed to be a promising tool for peptide transport studies and have been used to study the translocation mechanism of therapeutic peptides,^201,202^ cell-penetrating peptides^194,203,204^ and bacterial toxins.^205^

## 7. Imaging-based studies on cellular uptake and transport of peptides using single-cell and cell-layer systems

While unique insight into peptide-membrane interaction and translocation can be gained using GUV and GPMVs, they are simplified model systems which lack key biological features that are central to cellular uptake and transport, such as an energy dependent uptake machinery. Therefore, single-cell systems in combination with fluorescence microscopy have been a cornerstone for studying cellular transport of peptides.^17,59,206–208^ We note that such studies are compatible with many established and emerging imaging modalities (see Section 4.1) (Fig. 4, Single cell).

For some applications, conclusions may be made based on integrated intracellular levels of a fluorescently marked peptide and/or a rough cellular localization (with time-resolution) of peptides. In particular, such a simple data-analytic approach may be sufficient to provide evidence in favor of a particular peptide translocation mechanism^70–72,209^ when combined with proper control experiments, biophysical assays and/or model calculations. For example, either immediate cytosolic delivery, resulting from direct translocation across the membrane, or endocytic uptake of GFP was readily observed to depend on the cell-penetrating peptide conjugated to the green fluorescent protein (GFP).^210^ In this paradigm, one may maximize the temporal resolution of the imaging modality, since the signal from the fluorescent probes is typically not a limitation (see Section 4.1).

At the cellular level, mechanistic insight on uptake and translocation may be gained by the use of additional, organelle-specific markers that are spectrally separated from that of the peptide.^211,212^ The extent of co-localization is typically assessed using simple image correlations.^60^ Subcellular localization of peptides may also be revealed using other means, for example pH-sensitive probes.^211^ Such environmental dependencies of fluorophores may however be a caveat in co-localization analysis. In particular, fluorescence quenching may conceal co-localization of peptides with the membrane of cells and/or organelles, which, however, may be leveraged by diluting labeled peptide with its unlabeled analogue.^213,214^ In the presence of fluorescence quenching, care should be taken before integrated fluorescence intensities are used to quantify the density of peptides.

If peptide uptake in the cell does occur, fluorescence recovery after photobleaching (FRAP) can determine the mobile (free) and immobile (bound) fractions of molecules.^42,215,216^ In this approach, intense, focused light bleaches a well-defined spatial region of a sample followed by observation of the recovery of fluorescence with time as surrounding fluorophores diffuse into the bleached region. Diffusion coefficients of various diffusers are subsequently estimated by employing a theoretical model for diffusion of a (heterogeneous) population of molecules.^216^

Assays based on single cell models can provide important knowledge on the cellular uptake and transport of peptides.^217^ Simple intestinal barrier-like setups consisting of a confluent cell layer on a glass surface have added additional insights on barrier physiology^218,219^ and peptide/protein transport.^220^ However, neither single cells nor confluent cell layers on a glass surface recapitulate the complex three-dimensional characteristics and organization of fully differentiated cells making up an intestinal barrier. They fall short of accurately describing the chain of transport processes that the peptide experiences from one side of the intestinal barrier to the other in a polarized cell monolayer. This shortcoming makes physiologically relevant *in vitro* or *ex vivo* models of great interest, not only as end-point screening platforms for investigating peptide permeability, but also for dynamic in-depth mechanistic studies of peptide transport across the intestinal barrier. To facilitate such studies, sophisticated physiologically relevant models that are compatible with live cell imaging are emerging. The following sections review them in detail.

## 8. *In vitro* and *ex vivo* models for studying peptide translocation across intestinal barriers

### 8.1 Peptide transport and translocation studied using *in vitro* intestinal barrier models

**Fig. 7:**
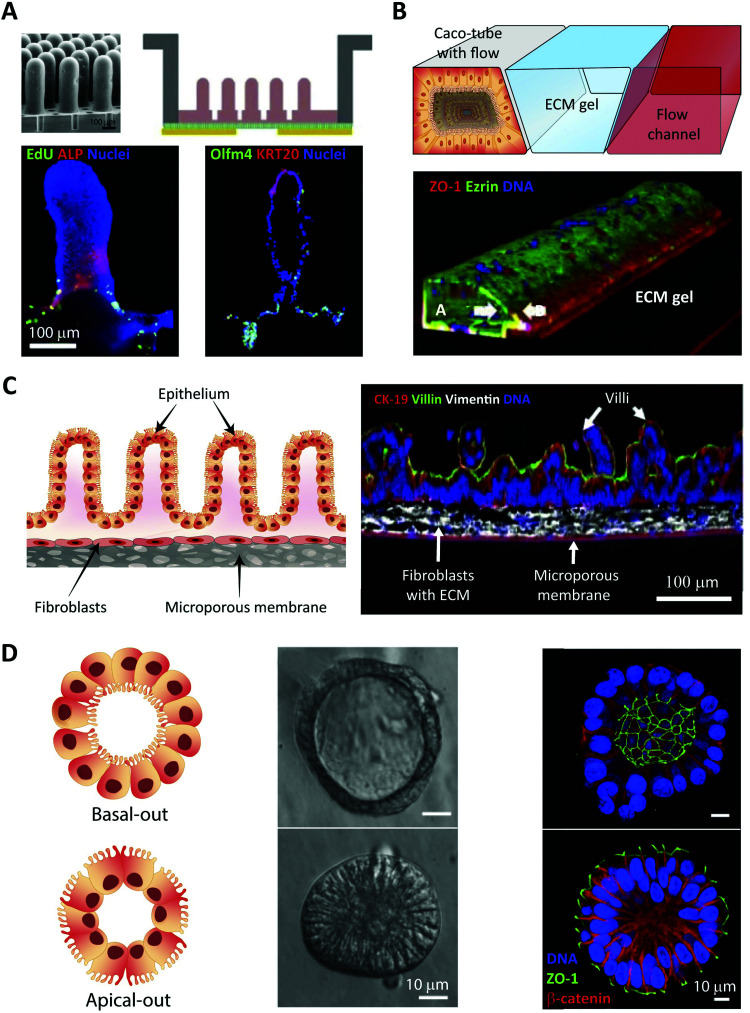
Summary of *in vitro* and *ex vivo* intestinal barrier models for studying peptide transport. (A) Example of micro-engineered scaffolds to generate crypt-villus architecture of human small intestinal epithelium. Top left, an electron microscopy image of the PDMS stamp used to create, top right, a micromolded collagen scaffold in a modified Transwell insert; bottom, visualization of cellular differentiation and polarization using immunostaining. Left, absorptive enterocytes localized on the villi (ALP, red) and proliferative cells localized in the crypt (EdU, green). Right, terminally differentiated tissues of the intestine expressing human cytokeratin 20 localized on villi tips (KRT20, red) and stem cells localized to crypts and adjacent regions (Olfm4, green). Reproduced from ref. 226 with permission from Elsevier, copyright 2017. (B) Top, OrganoPlate by MIMETAS: schematic of the three lane system at the center of each channel network, consisting of a Caco-2 cell tubular lane, an extracellular matrix (ECM) gel lane, and a perfusion lane. Bottom, 3D reconstruction of a confocal z-stack showing the Caco-2 cell tubular morphology visualized by staining the tight junction protein ZO-1 (red), the brush border-protein Ezrin (green), and DNA (blue). White arrows indicate the apical (A) and basal sides (B). Reproduced from ref. 232 with permission from Springer Nature, copyright 2017. (C) EpiIntestinal™ model, an *ex vivo* model for studying drug absorption in the small intestine based on primary human cell-based organotypic small intestinal micro-tissues. Left, an illustration showing different types of cells and the microporous membrane underneath. Right, immmunostained cross-sections of the reconstructed microtissues showing cytokeratin-19 stained columnar epithelial cells (CK-19, red), villin stained apical surface of epithelium (green) and vimentin stained fibroblasts in the underlying ECM substrate (white). Reproduced from ref. 235 with permission from Springer Nature, copyright 2018. (D) Left, two types of organoid morphology, basal-out and apical-out, can be produced, with the latter potentially facilitating studies of peptide transport across the intestinal barrier from the apical to the basal side. Middle, organoids imaged using modulation contrast microscopy. Right, confocal microscopy images with nuclei in blue, ZO-1 (green) and β-catenin (red) illustrate how the orientation of the organoid organization is flipped when going from the basal-out to the apical-out system. Reproduced from ref. 252 with permission from Cell Press, copyright 2019.

*In vitro* intestinal epithelial barrier models have traditionally been based on static 2D Transwell systems^221^ utilizing the human-derived adenocarcinoma cell line Caco-2 that spontaneously differentiates into small intestine-like enterocytes after 17–21 days (Fig. 4, monolayer). To generate a model that more closely resembles the *in vivo* scenario, goblet-like cells HT29 MTX^222^ and Raji B lymphocytes^223^ have been employed to induce mucus expression and to implement immunological features, respectively. Efforts have also been made to mimic the physiological villi-crypt structure of the small intestine by creating scaffolds made of porous poly(lactic-*co*-glycolic acid) (PLGA),^224^ micromolded collagen^225,226^ or silk fibroin proteins.^227^ As an example, culturing human small intestinal cells on a collagen-derived scaffold and creating a chemical- and growth factor gradient across the cell layer (Fig. 7A, top) allowed the formation of a differentiated and polarized cellular compartment mimicking aspects of the *in vivo* scenario (Fig. 7A, bottom).

The Transwell technology uses a microporous diffusion-open polymer membrane as cell culture support to enable exposure to difference chemical environments on opposing sides of a cell layer. It has been a vital platform for investigating intestinal absorption of peptides^228^ and has played a pivotal role in investigating passive paracellular/transcellular transport of peptides and the impact on their absorption profile upon various structural modifications.^229^ A major limitation of Transwell-based models is that they are not designed for microscopy and thus offer no direct visual insight on transport mechanisms (Fig. 1, left). The main obstacle is the distance between the cell monolayer and the basolateral chamber, which is not compatible with the working distance of high magnification, high numerical aperture objectives (see Section 4.1).^53^ Many studies circumvent this problem by chemically fixing the cell samples, excising the semi-permeable Transwell membrane, and placing it on a coverslip for imaging.^230^ This approach provides static snap-shots of drug transport, but it falls short of capturing the dynamics of cellular transport of peptides and drugs inside cells and across the barrier.^230^ In order to rectify these shortcomings, cells have been grown on the underside of the semi-permeable membrane, but the distance from the sample to the objective still remains a problem for high-magnification imaging.^53^ Other studies have made use of a small-volume, closed-bath imaging chamber that allows live imaging with higher-magnification objectives.^231^ However, this also requires a specialized microscope platform and stage adaptor, and the semi-permeable membrane must be cut out and placed in the chamber for imaging.^231^

### 8.2 Commercially available platforms for high-resolution live-cell imaging of peptide transport across intestinal barriers

More image-compatible cellular barrier systems than Transwells have been developed to facilitate high-end live cell imaging. The μ-Slide Membrane ibiPore Flow system (Ibidi, Germany) consists of two channels separated by a 0.3 μm-thick porous glass membrane and fluidic channels for inducing shear stress. The major advantage of this system is the highly transparent thin glass bottom (180 μm) and internal porous glass membrane, which gives access to real-time monitoring of drugs over time. However, the system is not designed for intestinal models and only allows liquid perfusion through the lower chamber. No intestinal transport studies have yet been reported with this system. Additional drawbacks of this single-chip system are the low throughput and the need for pumps, tubing systems, and related specialized equipment. In a recent development, an *in vitro* intestinal barrier model was established in the OrganoPlate (Mimetas, the Netherlands), a pump-free, microfluidic extracellular-matrix-based platform (Fig. 4, Organoplate) (Fig. 7B).^232^ In forty parallel chips, Caco-2 cells differentiate into polarized, intact monolayers and form intestinal tubules. Due to continuous perfusion cell differentiation takes place in only 4 days.^232^ In addition to the fast differentiation time and the lack of a physical membrane, the OrganoPlate is designed with three adjacent channels instead of the stacked topology of traditional models like the Transwell. This and its glass bottom strongly facilitate high-end live-cell imaging.^232^

### 8.3 *Ex vivo* barrier models offer increased biological complexity

Attempts to better preserve and recapitulate the biological complexity of the intestinal barrier have been made by the use of *ex vivo* intestinal models (Fig. 4, Microtissue). The Ussing chamber equipped with intestinal human or animal tissue^233^ is one model that has been utilized vastly for determining drug permeability. However, the model suffers from short viability of the tissue segments, low-throughput, and incompatibility with live imaging.^233,234^ A high-throughput alternative to the Ussing chamber is the InTESTine system (TNO, the Netherlands) that allows monitoring of 96 excised porcine tissue barriers simultaneously but also suffers from short tissue viability and imaging incompatibility issues.^234^ The EpiIntestinal model (MatTek, MA) is a high-throughput, human primary cell-based, 3D microtissue model that can be kept in culture for up to a month (Fig. 7C).^235^ The model demonstrates a higher correlation to human *in vivo* drug absorption profiles than classical Caco-2 Transwell systems^235^ and has also been used in a multi-organ-chip system to recapitulate absorption.^236^ As the EpiIntestinal model expresses many of the enzymes and transporters of the small intestine, peptide transport studies in this model could offer biologically relevant mechanistic insight. One promising approach for live-cell imaging of the model is 2PM, which can be employed for in-depth imaging of tissue samples, as explained in Section 4.1 (Fig. 3). The limitation of this approach is the compromise made on speed of imaging, resulting in limited information gained on dynamic intracellular processes. In addition to the more high-throughput solutions described above, the use of excised and fixed tissue sections for studying barrier transport is gaining more widespread use. Examples of this include the two recently FDA approved peptide formulations oral semaglutide (Section 2.3) and octreotide (Section 2.4).^29,33^ Tissue fixation allows for the use of immunostaining, and tissue clearing greatly increasing the ability to image deep into tissue with high spatial resolution.^237^ The main drawback of fixing excised tissue sections is the inability to perform live imaging and the potential artifacts introduced during fixation. The whole field of *ex vivo* barrier models is greatly benefitting from the emerging approaches enabling in-depth live imaging in tissues such as LLSM (see also Section 4.1) (Fig. 3). In combination with clearing techniques and adaptive optics, it has the possibility to revolutionize imaging deep into tissue samples with previously unseen levels of spatial and temporal resolution.^238^

Organoids are used as another tissue-mimicking model of the intestine, which is receiving increased interest in the field of drug delivery.^239^ Organoids are multicellular organotypic 3D-clusters generated from either primary tissue, embryonic tissue, or induced pluripotent stem cells, closely resembling the structural build seen *in vivo* (Fig. 7D).^240–242^ Organoids exhibit tissue-specific markers, with a self-renewal ability making them a complex and sophisticated model that is able to mimic a variety of organs (brain, liver, lung, gut, kidney, pancreas, and salivary gland). To date, however, intestinal organoids have primarily been used as disease models, for studying morphogenesis, or to investigate inflammatory host–pathogen interactions.^243–245^ Thus, the use of organoids in the context of analyzing peptide trafficking across a cellular barrier have been limited, predominantly due to the traditional basolateral-side out orientation of intestinal organoids resulting in inaccessibility of the apical side.^240^ To overcome this limitation, attempts have been made to use, *e.g.*, microinjections^246,247^ or to dissociate intestinal organoids into single cells and culture them as 2D-monolayers in classical Transwells^248,249^ or in more advanced organ-on a-chip systems.^250,251^ Another approach to gain access to the apical side, while aiming at maintaining the 3D identity of intestinal organoids, rely on inverting organoid polarity by modifying the culturing conditions, resulting in so called apical-out organoids.^252^ While these attempts show great potential, organoids must be fully verified as physiological relevant transport models, before they can emerge as a state-of-the art model system for studying peptide transport.

## 9. Imaging-compatible organ-on-a-chip microfluidic models

**Fig. 8:**
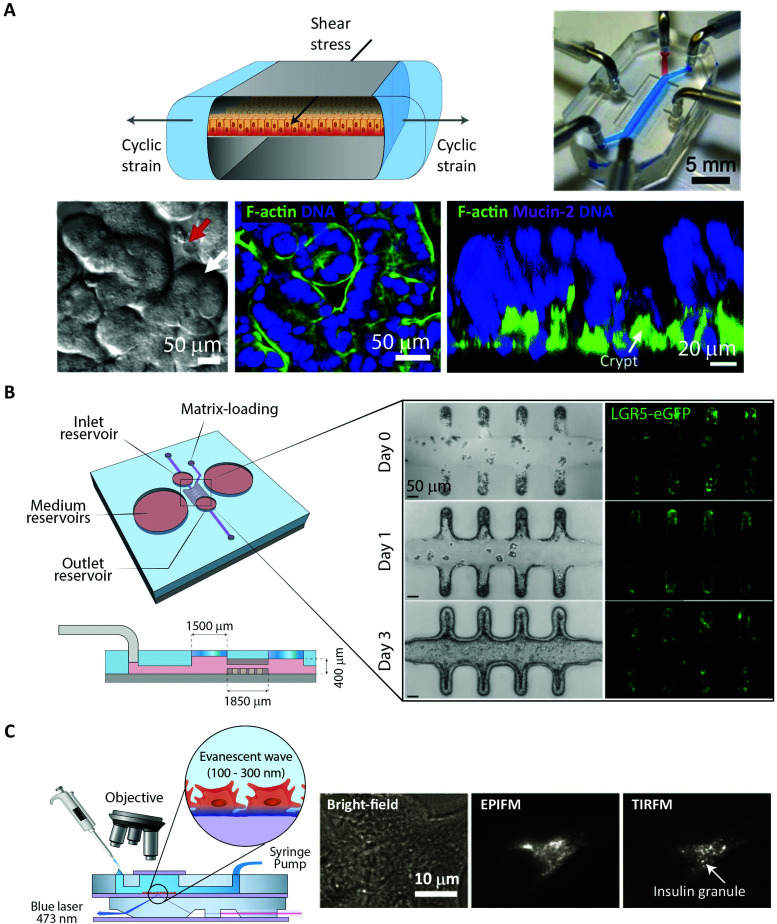
Imaging cells in lab-on-a-chip platforms. (A) Schematic cross-section of a microfluidic organ-on-a-chip. Top, left, device showing cyclic mechanical strain using the two vacuum chambers, which creates a shear stress in the perpendicular direction. Top, right, photograph of the device with blue and red dyes representing upper and lower microchannels, respectively. Bottom left, a differential interference contrast micrograph showing intestinal crypt (red arrow) and villi (white arrow) formation for Caco-2 cells grown in the chip. Bottom center, confocal immunofluorescence image of horizontal cross-section of intestinal villi stained for F-actin (green) labelling the apical brush border of polarized intestinal epithelial cells and DNA (blue), reproduced from ref. 268 with permission from National Academy of Sciences, copyright 2016. Bottom right, confocal fluorescence image showing vertical cross section of an intestinal villi inside the chip, stained for F-actin (green), Mucin-2 (magenta), and DNA (blue). Reproduced from ref. 256 with permission from The Royal Society of Chemistry, copyright 2012. (B) Schematic of microfluidic platform of the mini-intestine, consisting of a hydrogel chamber in the center, fed by the two medium reservoirs, perspective and side view (left). Bright-field and fluorescence time-course experiments showing the real-time formation of epithelium in mini-intestine chip (right). Reproduced from ref. 244 with permission from Springer Naturee, copyright 2020. (C) Left, schematic of the integrated device for observation of insulin granules inside the adherent cells cultured under continuous medium perfusion. Right, microscopic images of the MIN6-m9/insulin-GFP cells captured on the integrated system with different illumination modes, bright-field, epifluorescence microscopy (EPIFM), and TIRFM. Reproduced from ref. 284 with permission from Springer Nature, copyright 2012.

The most technically advanced cell-barrier models are the emerging organ-on-a-chip systems (Fig. 4, Organ-on-a-chip).^253,254^ In these, researchers combine tissue engineering and lab-on-a-chip technology to create a platform that allows accurate and biologically relevant studies of both cellular barrier physiology and peptide transport. In recent years, micro-engineered devices have been used to establish tissue models that mimic physiological function and structural complexity of human organs such as lung,^255^ intestine,^256,257^ liver^258,259^ and heart^260,261^*in vitro* (Fig. 8A). The intestine focused organ-on-a-chip systems represent minimal functioning units of the biological barrier providing a more native-like microenvironment within a micrometer-sized chamber as compared to their conventional counterparts, like Transwell- and 3D models (see Section 8.1). A major drawback of Transwell models is the absence of flow, which is an intrinsic feature of the native biological environment. With conventional 3D models, on the other hand, it is problematic to quantify transcellular transport, since sampling from the luminal content is challenging.^262,263^ Additionally, these systems lack tissue–tissue interfaces, such as vascular endothelium and parenchymal cells.^254^ The intestine-focused organ-on-a-chip systems combines living cells and continuous flow, creating a 3D model that exhibits key hallmarks of native tissues. Consequently, they offer the potential for more biologically accurate readouts of, *e.g.*, peptide transport across cellular barriers.

One strategy to recreate the multicellular interface of organs is to incorporate polymer membranes into the microfluidic channel of these devices.^264–269^ Most commonly, poly(dimethylsiloxane) (PDMS) is used as fabrication material for such systems. PDMS is transparent and thus enables high-resolution microscopy of the developed intestinal barrier model.^256,257^ This strategy has been widely employed to develop gut-on-a-chip systems and is popular among the researchers in the field. Another interesting approach uses microfluidic hydrogels with built-in microchannels, which are intrinsically highly permeable to biomolecules and therefore alleviate the need for micropores.^270–272^ Moreover, microfabrication techniques, specifically 3D-printing, offer the possibility of creating complex microchannel networks and microarchitectures through a one-step procedure.^271,273–275^ Recently the above-mentioned technologies were combined to develop a hybrid mini intestine, composed of an elastomeric PDMS-based frame and a hydrogel compartment for cell culture.^244^ Exploiting the self-organization property of the intestinal stem cells, a tubular epithelium with similar spatial arrangement of crypt- and villus-like domains was generated as well as an accessible lumen. Horizontal orientation of the device—like most of the gut-on-a-chip systems in general – further facilitates monitoring of intercellular translocation by means of high-resolution imaging.^244,276–278^ In contrast to other off-the-shelf solutions, such as living organs or macroscale 3D models, the chip-based models offer unique possibilities for high-resolution, real-time imaging of biological phenomena at the molecular scale within a 3D tissue model.^279,280^ More specifically, integration of the mini-intestine chip with state-of-the-art microscopes and cameras enabled bright-field and fluorescence live imaging of the intestinal tissue development, regeneration, and parasite infection (Fig. 8B). Another interesting example of high-resolution imaging of organ-on-a-chip models is the implementation of stochastic optical reconstruction microscopy (STORM, see Section 4.3) on a simple and versatile microfluidic platform to visualize mitochondrial protein distribution in live cells.^281^

Besides cell orientation, the microfluidic platforms present several other advantages over their classical counterparts: They facilitate the required long-term high-resolution image acquisition by providing stable conditions for the cells, and, at the same time, they enable monitoring of cell growth and division.^244,282^ This advantage has been utilized in the development of an integrated microfluidic device capable of real-time imaging of living cells with high signal-to-noise ratio under continuous perfusion.^283^ Using TIRFM (see Section 4.1), the setup allows non-invasive *in situ* of the location of insulin granules in mouse pancreatic β-cells (Fig. 8C).^284^ This is to date the most relevant study where advanced microscopy and organ-on-a-chip have joined forces to provide valuable insights into a biological translocation. However, it must be noted that organ-on-a-chip technology is in its infancy and many tissue characteristics still need to be engineered and integrated in a robust format to reach broad application. As mentioned, these microfluidic devices^256,257,285–289^ have been successfully coupled with imaging setups for real-time imaging, which makes them candidates for providing detailed mechanistic understanding of complex biological phenomena, such as peptide transport across the intestinal barrier.

The organ-on-a-chip technology is rapidly growing. However, from a technological standpoint, several overarching considerations and operative challenges must be tackled to fully realize the potential of these micro-engineered devices and successfully translate them from proof-of-concept to practical screening platforms. One of the very basic issues arises from the material, PDMS, which is the most commonly used for fabrication. Albeit allowing high-resolution imaging, PDMS absorbs small hydrophobic compounds, including drug molecules,^290,291^ which may hinder accurate evaluation of the parameters of interest. Furthermore, lack of a standardized automated fabrication process poses additional technical hurdles in terms of high-throughput operation and reproducibility. Another underdeveloped aspect of on-chip assays is the fact that high-end fluorescence imaging still relies mainly on fixed and immunostained samples. This is an end-point analysis, while current live-imaging techniques used in organ-on-a-chip platforms are mostly still limited to WFM modes, such as bright-field and epifluorescence imaging (see Section 4.1). Looking forward, one can envision highly automated systems integrating microfluidic devices, sophisticated built-in sensors,^292–294^ and advanced multiplex imaging techniques.

## 10. *In vivo* imaging of transport across the intestinal barrier

**Fig. 9:**
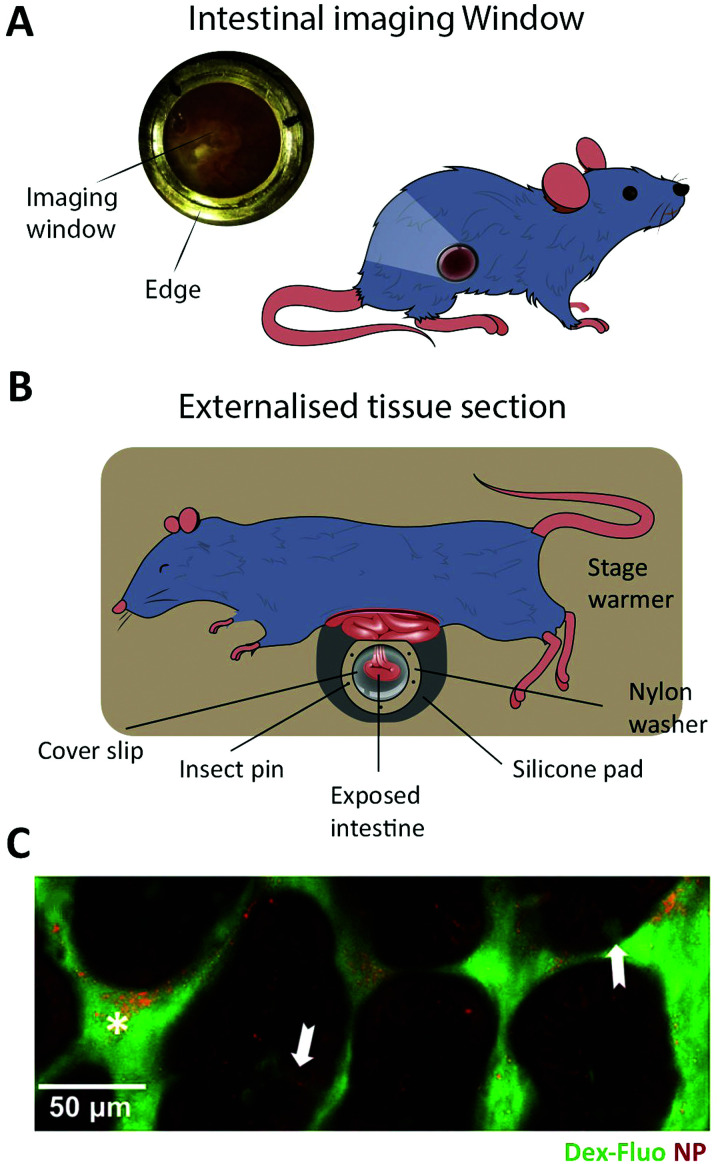
*In vivo* imaging of the intestinal barrier. (A) An illustration showing a mouse with a surgically implanted imaging window allowing visualization of the small intestine. (B) A schematic showing an IVM setup where an intestinal section is externalized from an anesthetized mouse and placed inside a glass based imaging chamber. (C) An example of a IVM setup being used to demonstrate differential uptake of orally administered particles and antigens. Dextran-fluorescein (Dex-Fluo) (green) enters through Goblet cell-associated passageways (white arrows), while 20 nm polystyrene nanoparticles (NP) (red) enter *via* intestinal epithelial cells. Reproduced from ref. 303 with permission from Public Library of Science, copyright 2014.

Despite the immense amount of insight gained on the fundamental function of cellular barriers using *in vitro* and *ex vivo* models of various complexity, such systems are inherently limited and do not fully recapitulate the natural *in vivo* milieu. Consequently, within the last few decades, various *in vivo* imaging platforms have been developed.^295,296^ They allow for continuous fluorescence imaging of a plethora of different living tissues, including the intestinal cellular barrier. Such intravital microscopy (IVM) setups allows for long-time measurements, ranging from hours to days, under minimally invasive conditions (Fig. 4, Intravital and Fig. 9). This circumvents the accelerated tissue degradation observed in, *e.g.*, explanted tissue models.^237,297^ In contrast to whole-body *in vivo* imaging modalities like MRI and PET-CT, which typically have a spatial resolution in the millimeter range,^298,299^ IVM allows for imaging with single-cell resolution, making it ideally suited for elucidating the molecular mechanisms governing peptide transport across cellular barriers.^299^ The single-cell resolution of IVM also allows for quantitative measures of cellular barrier heterogeneities with respect to both physiology and transport processes.^295,299,300^ The main limitations for employing IVM is the requirement for sophisticated equipment, both with respect to advanced microscopes and animal-handling setups, as well as specially trained and skilled personnel.^296,297^ These technical requirements and the tedious data acquisition mean that IVM inherently is not a high through-put technique. Instead it offers an unmatched level of detail and biological accuracy.^295^

To facilitate IVM of the intestinal barrier, one method relies on an intestinal section being externalized from an anesthetized mouse and placed inside a glass-based imaging chamber (Fig. 9A).^297^ The subcellular resolution provided by such setups has been used to determine *in vivo*, the molecular mechanism by which tumor necrosis factor α disrupts the intestinal barrier integrity through modification of TJ structure and function.^301^ For transport studies, both peptides and proteins have been imaged crossing the intestinal barrier. This has offered unique insights on how specific epithelial- and immune cells coordinate the transport process *in vivo* (Fig. 9A and C).^302,303^ In a drug-delivery context, the setup has been used to elucidate a clear dependency on NP size in both the amount and the route of transport across the intestinal barrier. Smaller particles displayed increased transport.^303^ Finally, IVM was used to visualize how NP-delivered insulin interacted with the microvilli of the intestine.^304^ The molecular mechanism of the insulin absorption was revealed to depend on a direct interaction with the apical sodium-dependent bile acid transporter.

IVM may also be performed in conjunction with a surgical implantation of an imaging window through which the microscopy can be performed^295,305^ (Fig. 9B). This methodology does not rely on tissue externalization and thus allows IVM to be performed on a larger range of cellular barriers. The main benefit of performing imaging window-based IVM is the ability to image tissue under near-native conditions and in its orthotopic position without this imaging being compromised by the detrimental optical distortions typically experienced when imaging non-superficial tissues.^306^ Additionally, the technical advances in surgical procedures and window implementation now make it possible to image cellular barriers for extremely long periods (up to months), while maintaining subcellular resolution.^295,307^ However, it is important to keep in mind that this technique requires highly specialized staff and sophisticated equipment. Also, it remains prone to trauma and contaminants introduced during the surgery and to damaging fluid accumulation over time. All of this limits the use of the technique to a restricted number of highly specialized groups.^307^ Recently, surgically implanted imaging windows in the mouse abdomen have allowed researchers to perform IVM of living intestinal tissue.^306^ This has facilitated novel insights on how the complex cellular architecture of the micro villi formations lining the intestinal barrier is produced and maintained.^300^ These methods are still in their infancy and have, to our knowledge, not been employed for studying peptide transport across the cellular barrier. We envision, however, that such studies should be feasible with current setups. Overall, the unique ability of IVM to study biological processes and drug transport in conditions resembling the native environment will make IVM an increasingly important tool for reducing the translational gap between early drug development and clinical efficiency.^308,309^

## 11. Peptide-based nanoparticle delivery systems

The main focus of this review is on the transport across cellular barriers of peptides as individual entities. However, another appealing mode of delivering peptide and proteins is to assemble them into NPs. Here we define such peptide/protein NPs to be structures where the core element is the peptide/protein itself and thus not delivery vehicles where peptides/proteins are, *e.g.*, encapsulated. For a thorough discussion on the latter type of delivery system see the recent review by Brayden *et al.*^21^ Making peptide/protein NPs can be done either through self-assembly, chemical cross linking, de-solvation, or a combination of approaches.^310^ The benefits of NPs include: protection of the peptides/proteins against enzymatic degradation,^310^ improved physicochemical properties compared to free peptide/protein, increased cellular uptake, reduced clearance from tissue microenvironments, the ability to bypass biological barriers (*e.g.*, mucus^8^ and glycocalyx^311^), as well as the potential to deliver a high payload of biologically active molecules. The transport across intestinal barriers of NPs is highly dependent on the physicochemical properties of the particle.^312,313^ Larger NPs (100–200 nm) are commonly endocytosed by clathrin- or caveolin-mediated endocytosis, while smaller (<100 nm in diameter) particles can be taken in by macro- or micropinocytosis. Some protein NPs will be endocytosed by interacting with the receptor of the free protein on the cell surface. For instance, albumin particles such as Abraxane, a 130 nm diameter bovine serum albumin particle loaded with the chemotherapeutic paclitaxel, has been shown to interact with the endothelial gp60 receptor to induce caveolin mediated endocytosis (similar to free albumin).^314^ Unzueta *et al.* showed that 13 nm self-assembled NPs from GFP coupled to the peptide T22 interact with the CXCR4 receptor and lead to uptake into endosomes.^315^ Interestingly, they found that the T22 peptide bound more weakly to the receptor than other peptides tested but that the T22-GFP NP was the only construct that lead to cellular uptake.^315^ More detailed imaging might lead to a deeper understanding of the reason for the discrepancy between receptor-binding affinity and cell uptake. Estrada *et al.* also used GFP to formulate NPs. These chemically crossed-linked particles carrying β-galactosidase showed protection of the active enzyme in the biological environment as well as increased cellular uptake of the enzyme when delivered by the 283 nm negatively charged protein particles and compared to free enzyme in solution.^316^ GFP has also been used to show protein encapsulation into Qβ-virus-like protein particles that helped to protect the encapsulated protein against thermal denaturation and degradation by proteases and lead to efficient uptake in CD22^+^ cells.^317^

However, cellular uptake is not enough to cross epithelial barriers such as the ones found in the intestine, and knowledge about particle exocytosis and transcytosis is crucial.^318^ Bramini *et al.* showed, using fluorescently labeled polystyrene NPs, how a Transwell setup and a combination of CLSM and TIRFM (see Section 4.1) (Fig. 3) can be used to evaluate transport of NPs through a tight epithelial barrier.^319^ It would be interesting to see methods like these applied to peptide/protein particle systems, such as the ones described above, to gain more mechanistic insights on the transport and biological fates of these biodegradable systems after endocytosis. Different imaging techniques can also be utilized to evaluate NP interaction with mucosal barriers, such as particle tracking (see Section 4.2).^320^ Surface charge of NPs has shown to have great impact on NP diffusion in mucus, with negatively and neutrally charged particles being able to diffuse through mucus while positively charged particles get trapped.^320^ This interaction can possibly be utilized to ensure prolonged residence time of a NP system in the intestine allowing slow release of single proteins or peptides.^321^ There are numerous examples of such prolonged NP interactions with biological barriers facilitating better delivery of vitamins^313,322^ and hydrophobic drugs^323–325^ and a few for delivery of active protein and peptides.^316,326^ These highly biocompatible systems show great potential for improved drug targeting, availability, and enhanced patient compliance.

## 13. Conclusion

Poor predictive power of current preclinical drug testing results in major failures in clinical trials and consequently in high costs of research and development in the pharmaceutical industry.^308,327^ To remedy this, it has been proposed to enhance the focus on understanding the biological mechanisms that facilitate drug uptake and transport.^9,12^ This focus requires new barrier models and technical methods. Fluorescence imaging is poised to be part of the solution, and currently there is a rapid development of advanced intestinal barrier models that are compatible with high-end imaging modalities. This progress in model development coincides with the increased access to (L)LSM setups, both offering the imaging speed, sensitivity and low phototoxicity needed to implement 4D microscopy as a stable in peptide translocation studies. Here, we have provided an overview of the various aspects and possibilities one should consider before engaging in studies of peptide transport across the intestinal barrier using fluorescence microscopy. Overall, the endeavors reviewed here pave the way for cutting-edge, dynamic, high-resolution mechanistic transport studies in highly sophisticated barrier models that recapitulate the physiological features of the small intestine with enhanced precision. This should result in improved end-point efficacy in oral delivery and thereby bridge the translational gap between bench and bedside for peptide biopharmaceuticals.

## Author contributions

Wrote or contributed to the writing of the manuscript: J. B. Larsen, Taebnia, Dolatshahi-Pirouz, Eriksen, Hjørringgaard, Kristensen, N. W. Larsen, N. B. Larsen, Marie, Mündler, Parhamifar, Urquhart, Weller, Mortensen, Flyvbjerg, Andresen.

## Conflicts of interest

None.

## Supplementary Material
